# Structural insights into proteolytic activation of the human Dispatched1 transporter for Hedgehog morphogen release

**DOI:** 10.1038/s41467-021-27257-w

**Published:** 2021-11-29

**Authors:** Wanqiu Li, Linlin Wang, Bradley M. Wierbowski, Mo Lu, Feitong Dong, Wenchen Liu, Sisi Li, Peiyi Wang, Adrian Salic, Xin Gong

**Affiliations:** 1 Department of Biology, School of Life Sciences, Southern University of Science and Technology, 518055 Shenzhen, Guangdong China; 2 Department of Cell Biology, Harvard Medical School, Boston, MA 02115 USA; 3 SUSTech Cryo-EM Facility Center, Southern University of Science and Technology, 518055 Shenzhen, Guangdong China; 4 Present Address: Department of Pharmacology, School of Medicine, Southern University of Science and Technology, 518055 Shenzhen, Guangdong China; 5 Present Address: Department of Biochemistry and Molecular Biology, International Cancer Center, Shenzhen University Health Science Center, 518060 Shenzhen, Guangdong China

**Keywords:** Membrane proteins, Morphogen signalling, Cryoelectron microscopy

## Abstract

The membrane protein Dispatched (Disp), which belongs to the RND family of small molecule transporters, is essential for Hedgehog (Hh) signaling, by catalyzing the extracellular release of palmitate- and cholesterol-modified Hh ligands from producing cells. Disp function requires Furin-mediated proteolytic cleavage of its extracellular domain, but how this activates Disp remains obscure. Here, we employ cryo-electron microscopy to determine atomic structures of human Disp1 (hDisp1), before and after cleavage, and in complex with lipid-modified Sonic hedgehog (Shh) ligand. These structures, together with biochemical data, reveal that proteolytic cleavage opens the extracellular domain of hDisp1, removing steric hindrance to Shh binding. Structure-guided functional experiments demonstrate the role of hDisp1–Shh interactions in ligand release. Our results clarify the mechanisms of hDisp1 activation and Shh morphogen release, and highlight how a unique proteolytic cleavage event enabled acquisition of a protein substrate by a member of a family of small molecule transporters.

## Abstract (web summary)

Dispatched (Disp) RND transporter, activated by Furin-mediated proteolytic cleavage, mediates the release of the lipid-modified Hedgehog (Hh) ligands. Here, the authors report structures of human Disp1 (hDisp1) before and after cleavage, and in complex with lipid-modified Sonic hedgehog (Shh), with insights into the mechanisms of hDisp1 activation and function.

## Introduction

The Hedgehog (Hh) signaling pathway is involved in orchestrating embryonic development and tissue homeostasis, and its dysregulation leads to various human diseases, including cancer and congenital malformations^1–5^. The pathway is activated by the secreted Hh ligand, which is synthesized as a longer precursor that undergoes autoproteolytic cleavage catalyzed by its C-terminal intein domain (Hh-C), to generate an N-terminal domain (Hh-N) covalently attached to cholesterol at its C-terminus^6^. Hh-N is further palmitoylated on its N-terminus by the Hh acyltransferase, Hhat^6–9^, thus generating the mature Hh ligand. The hydrophobicity imparted by this unique dual lipidation causes Hh ligands to be firmly attached to the plasma membrane of producing cells; however, during development, Hh ligands are released and spread extracellularly, to signal to target cells located many cell diameters away. Release of lipid-modified vertebrate Hh ligands, such as Sonic hedgehog (Shh)^10,11^, relies on a dedicated transport system involving the transmembrane protein Dispatched (Disp) and a member of the Scube family of soluble extracellular chaperones^12–14^. Disp and Scube act cooperatively, with Disp catalyzing transfer of Shh from the membrane to the Scube acceptor^15^, through a postulated hand-off mechanism^14^, ensuring that the lipid appendages of Shh are shielded from the aqueous environment. Subsequently, the secreted Scube–Shh complex diffuses extracellularly and delivers Shh to the surface of target cells^16^, where it ultimately binds its membrane receptor, Patched (Ptch), initiating signal transduction^17–19^.

Disp belongs to the RND superfamily of small molecule transporters^20^, which includes prokaryotic members such as AcrB^21^ and HpnN^22^, and eukaryotic members such as Ptch1^23–28^ and the lysosomal Nieman-Pick type C disease protein 1 (NPC1)^29,30^. All members of the RND family except Disp transport small molecule substrates: AcrB transports antibiotics and other toxicants, HpnN transports hopanoids, while Ptch1 and NPC1 transport cholesterol. Disp is unique among RND proteins in that its substrate, the dually lipidated Hh ligand, is a protein. Thus, a critical unanswered question is how Disp acquired the ability to transport a macromolecule, rather than a small molecule.

Also uniquely among RND proteins, Disp activity requires Furin-mediated cleavage at a conserved site in its first large extracellular domain (ECD1)^31^, yielding a mature Disp protein consisting of two non-covalently associated fragments. Blocking cleavage alters Disp membrane distribution in polarized cells, and greatly reduces Hh ligand release, thus inhibiting Hh signaling^31^. It was proposed that cleavage regulates Disp maturation and function^31^, but the mechanism by which this proteolytic event leads to Disp activation remains unknown.

Recently, cryo-EM structures of *Drosophila* Disp (dDisp) and human Disp1 (hDisp1) were reported at 3.2 Å and 4.5 Å resolution, respectively^32,33^, as well as low-resolution cryo-EM structures of dDisp and hDisp1 in complex with Hh ligands [4.8 Å and 7.9 Å resolution, respectively^32,33^]. In all these structures, the proteolytic state of Disp is unclear, thus they do not answer how cleavage affects Disp function. Furthermore, the mechanism of Disp-mediated Hh ligand release, and especially our understanding of how Disp transports a macromolecular substrate, have remained unclear.

Here, we use cryo-EM and functional experiments, to ask several key questions in Hh signaling: how does cleavage control Disp, how did Disp acquire a protein substrate, and what is the mechanism involved in Hh ligand release? We first solve cryo-EM structures of hDisp1 in pre- and post-proteolytic cleavage states, at overall resolution of 3.61 Å and 3.68 Å, respectively. The structures reveal that cleavage causes opening of the two halves of the large extracellular domain of hDisp1 (ECD1 and ECD2) and removes a steric block posed by an unstructured loop, which allows cleaved hDisp1 to bind Shh with greatly increased affinity. We next solve the cryo-EM structure of hDisp1 in complex with the native, dually lipidated Shh ligand, at an overall resolution of 4.07 Å, and we use structure-guided experiments to demonstrate the role of hDisp1–Shh interactions in Shh release from producing cells. Our results elucidate how cleavage activates the ability of Disp to transport Hh ligands, and clarify the mechanism of ligand release, an essential step in the Hh signaling pathway.

## Results

### Purified hDisp1 is partially processed by proteolytic cleavage

**Fig. 1:**
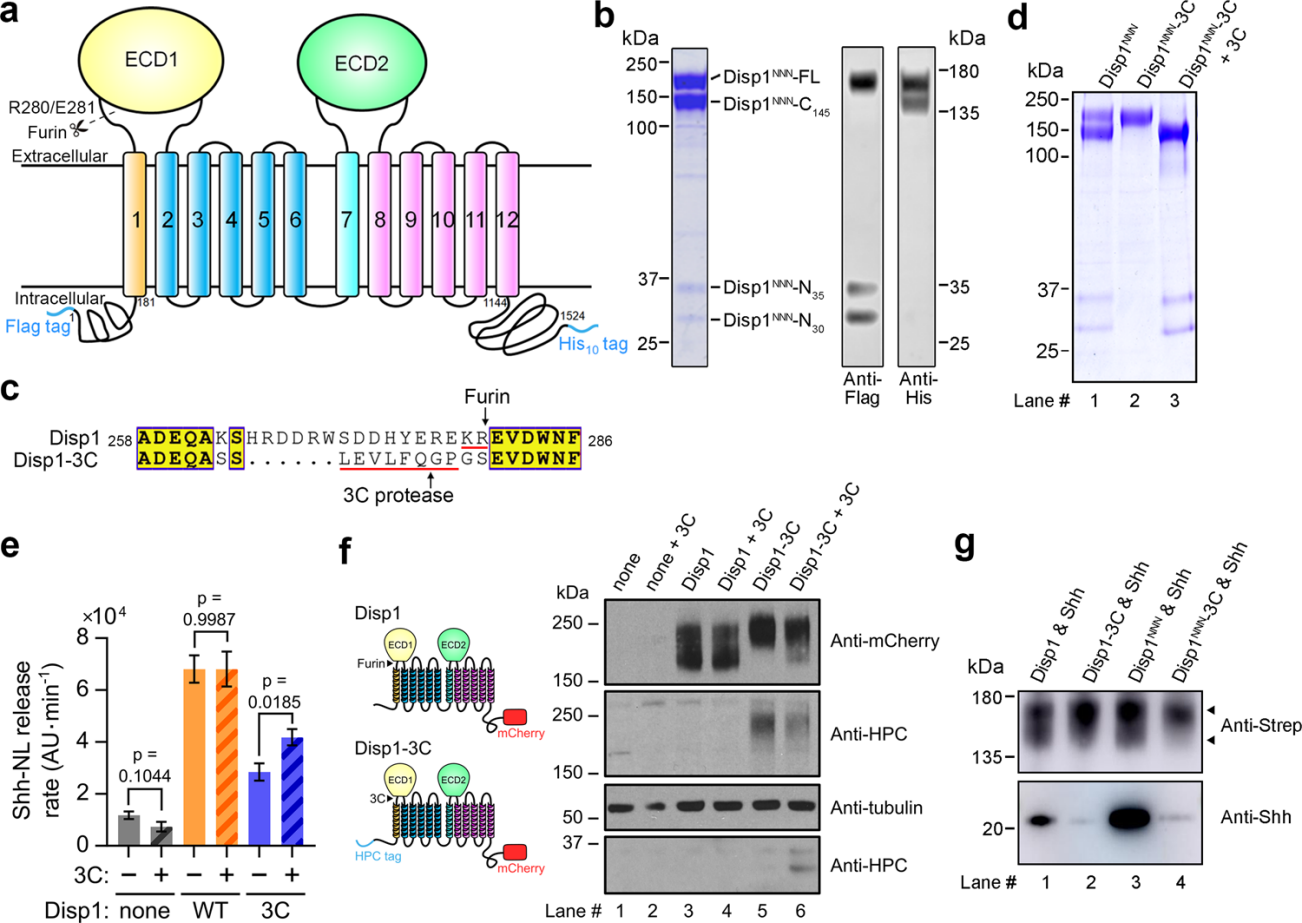
Purification of human Disp1 (hDisp1) in pre- and post-proteolytic cleavage states. **a** Topological diagram of hDisp1. Disp homologues contain 12 transmembrane segments (TMs), two large extracellular domains (ECD1 and ECD2), as well as flexible N- and C-terminal intracellular domains. TMs 2-6 constitute a conserved sterol-sensing domain (SSD), found in various membrane proteins related to cholesterol transport and metabolism, including Ptch, NPC1, and the endoplasmic reticulum cholesterol sensor SCAP^56^. The N-terminal Flag tag, C-terminal His_10_ tag, and the predicted Furin cleavage site between Arg280 and Glu281 are indicated. **b** Purified hDisp1^NNN^ was separated by SDS-PAGE, and was detected by Coomassie staining (left) or Western blot (right). Full-length and cleavage products are indicated. Purified hDisp1^NNN^ consists of a mix of cleaved and uncleaved species. **c** The Disp1-3C construct, in which the Furin cleavage site is replaced by a 3C protease cleavage site. **d** Purified hDisp1^NNN^-3C consists entirely of uncleaved protein (lane 2), which is quantitatively cleaved by incubation with 3C protease (lane 3). Purified, partially Furin-cleaved hDisp1^NNN^ is shown for comparison (lane 1). **e** Disp1-3C supports Shh release from cells in cleavage-dependent manner. Wild-type (WT) hDisp1 or hDisp1-3C was stably co-expressed with NanoLuc-tagged Shh (Shh-NL) in Disp1-null HEK293T cells. The cells were incubated with purified recombinant mouse Scube2 (1 μM) in serum-free media, in the presence of the protein synthesis inhibitor, cycloheximide (100 μg/mL). Time course of background-subtracted Shh-NL release was measured for six time points in a single independent biological experiment, by NanoLuc luminescence. Initial rates of Shh-NL release were normalized to Shh-NL expression levels in each cell line. Bars represent best-fit slope of a linear regression fit to the release time points, and error bars represent standard error of the regression fit. Shh release is reduced for Furin-uncleavable Disp1-3C compared to WT Disp1, which is cleaved by Furin. ANCOVA was used for testing for differences in release rate for each Disp1 variant upon 3C protease addition. Pretreating the cells with 3C protease enhances Shh-NL release in Disp1-null cells rescued with hDisp1-3C (*p* = 0.0185), but not in unrescued cells (*p* = 0.1044), or cells rescued with WT hDisp1 (*p* = 0.9987). **f** Immunoblot for the experiment in (**e**), showing Disp1-3C cleavage by exogenously added 3C protease. Both WT hDisp1 and hDisp1-3C are tagged with mCherry at the C-terminus, and hDisp1-3C is also tagged with HPC at the N-terminus, as indicated in the diagram to the left. Note the 3C protease-dependent appearance of an N-terminal fragment of hDisp1-3C. **g** Pull-down assay showing that uncleaved hDisp1-3C and hDisp1^NNN^-3C exhibits greatly reduced binding to Shh compared to hDisp1 and hDisp1^NNN^, which are cleaved. The WT and mutant hDisp1 constructs were co-expressed with Shh in HEK293F cells, and immunoprecipitated hDisp1 and Shh were analyzed by Western blotting. Source data for (**b**) and (**d**–**g**) are provided as a Source Data file.

To obtain protein suitable for structural studies, we first attempted to express full-length (FL) wild-type (WT) hDisp1 (Fig. 1a) in HEK293F cells. This protein was poorly expressed, so we surveyed a series of other hDisp1 constructs. Ultimately, a triple-point mutant (hereafter hDisp1^NNN^) in which three conserved aspartate residues located in transmembrane helices TM4 and TM10 (Asp572, Asp573, and Asp1051) are mutated to asparagine, showed sufficient expression and was well behaved biochemically after purification in detergent (Supplementary Fig. 1a); this Disp1 mutant is known to bind Shh stronger than WT Disp1^14^. Consistent with reports of Disp1 oligomerization^31,34^, analysis of our hDisp1^NNN^ preparations by size-exclusion chromatography (SEC) showed that a portion of the protein exists as oligomers (Supplementary Fig. 1a). However, the oligomeric species appeared heterogeneous by cryo-EM, so we focused our analysis on the monomeric form of hDisp1^NNN^ (see below).

Purified hDisp1^NNN^ migrated on SDS-PAGE as two larger bands with apparent molecular weights of ~175 and ~145 kDa, and two smaller bands with apparent molecular weights of ~35 and ~30 kDa (Fig. 1b, left panel). Western blotting indicated that the 175 kDa band corresponds to FL hDisp1^NNN^, as it contained both the N-terminal Flag tag and C-terminal His tag, the 145 kDa band corresponds to a cleaved product with only the C-terminal His tag (hDisp1^NNN^-C_145_), while the two smaller bands correspond to cleaved products with only the N-terminal Flag tag (hDisp1^NNN^-N_35_ and hDisp1^NNN^-N_30_) (Fig. 1b, right panel). This cleavage pattern of hDisp1^NNN^ is consistent with the recently reported partial cleavage of mouse and *Drosophila* Disp proteins by Furin protease^31^. Moreover, hDisp1^NNN^ and WT hDisp1 displayed the same proteolytic processing pattern when expressed and purified from the HEK293F cells (Supplementary Fig. 1b), indicating that hDisp1^NNN^ is a suitable construct for structural investigations of how proteolytic processing affects hDisp1.

### Purification of hDisp1 in distinct proteolytic cleavage states

Furin cleavage is critical for Disp1 function in vivo, but how this event activates Disp1 remains unknown. To determine the effect of Furin cleavage on hDisp1 structure, we wanted to compare the structure and properties of hDisp1 before and after proteolytic cleavage. We thus developed a strategy to obtain pure uncleaved and cleaved hDisp1 preparations. To this end, we replaced the region in hDisp1 recognized by Furin (residues 263–280) with a cleavage site for the highly specific 3C protease (Fig. 1c), and we purified to homogeneity the resulting hDisp1^NNN^-3C protein (Supplementary Fig. 1c). Purified hDisp1^NNN^-3C migrated as a single band on SDS-PAGE, of the same molecular weight as FL hDisp1^NNN^ (Fig. 1d). Notably, the hDisp1^NNN^-3C preparation was devoid of the low molecular weight species found in purified hDisp1^NNN^ (Fig. 1d), indicating that Furin cleavage had been successfully abolished. Importantly, upon incubation with 3C protease, hDisp1^NNN^-3C was quantitatively cleaved into a larger band and two smaller bands, similar to purified hDisp1^NNN^ (Fig. 1d); this indicates that our hDisp1^NNN^-3C construct is suitable for generating pure hDisp1 in either cleaved and uncleaved state.

We asked if the introduction of the 3C protease cleavage site preserves hDisp1 activity and regulation by proteolytic processing, by assaying hDisp1-3C ability to rescue Scube-dependent Shh release from hDisp1-null cells. To this end, we used a fast and sensitive assay for measuring Shh release kinetics^15^, based on NanoLuc luciferase (NL)-tagged Shh (Shh-NL). As shown in Fig. 1e, in the absence of 3C protease treatment, hDisp1-3C supported a slower Shh-NL release rate from cells compared to WT hDisp1, consistent with the importance of proteolytic cleavage for hDisp1 function. Importantly, when cells were briefly treated with recombinant 3C protease, Shh-NL release by hDisp1-3C was specifically enhanced (Fig. 1e), indicating that 3C protease-cleaved hDisp1-3C is functional in Shh release. We confirmed that 3C protease added to cells indeed caused specific cleavage of hDisp1-3C, by Western blot (Fig. 1f). We note that only a small fraction of hDisp1-3C was cleaved by brief treatment with exogenous 3C protease (Fig. 1f, lanes 5 and 6), in contrast to the efficient cleavage of WT hDisp1 by endogenous Furin (Fig. 1f, lanes 3 and 4); this is consistent with the modest enhancement of Shh release rate (Fig. 1e). Furthermore, in the continued presence of purified 3C protease, hDisp1-3C enhanced Shh release relative to mock-transfected hDisp1-null cells, confirming that cleaved hDisp1-3C is functional (Supplementary Fig. 1d). Thus, hDisp1-3C recapitulates cleavage-dependent activation in promoting Shh release from cells.

Finally, we asked how proteolytic cleavage might be required for hDisp1 activity. One possibility is that cleavage affects the interaction between hDisp1 and Shh. Although hDisp1^NNN^ is inactive in Shh transport^15,35^, it binds Shh stronger than WT hDisp1^14^ (Fig. 1g, lanes 1 and 3). As shown in Fig. 1g, uncleaved hDisp1-3C and hDisp1^NNN^-3C showed greatly reduced binding to Shh compared to hDisp1 and hDisp1^NNN^ in an affinity pull-down assay, suggesting that the interaction between hDisp1 and Shh is cleavage-dependent for both WT hDisp1 and hDisp1^NNN^. Thus, cleavage is important for hDisp1 activity, at least in part by controlling Shh binding (see below and Discussion).

### Proteolytic cleavage causes conformational change in hDisp1

**Fig. 2:**
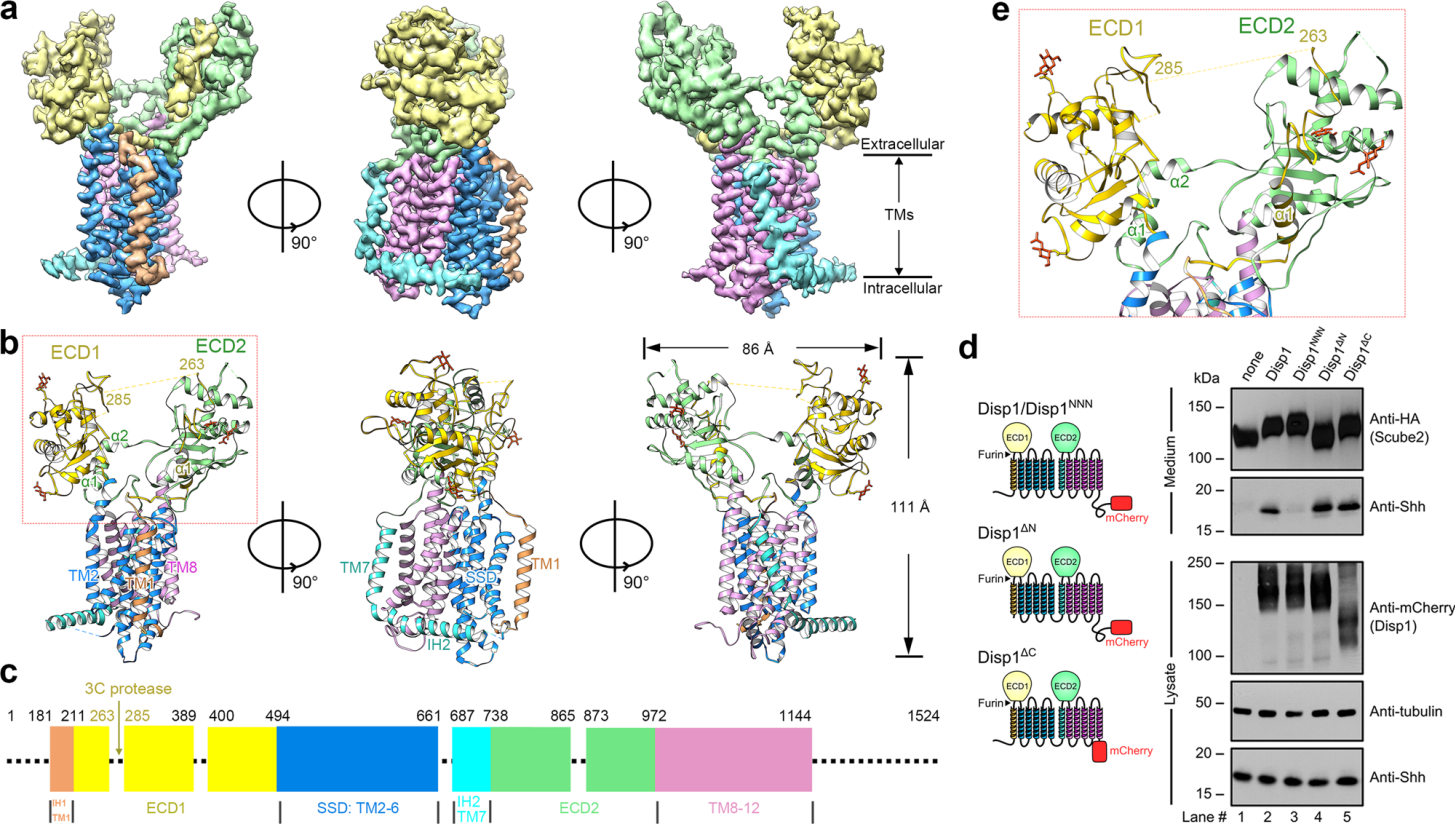
Structure of hDisp1 in uncleaved state. **a**, **b** Corresponding views of the cryo-EM density map (**a**) and atomic model (**b**) of uncleaved hDisp1^NNN^-3C. Domains are colored as follows: IH1 and TM1, orange; ECD1, yellow; TM2–6 (SSD), blue; IH2 and TM7, cyan; ECD2, green; TM8–12, pink. **c** Sequence coverage of the atomic model and annotation of the sequence. Dashed lines indicate portions of the protein that were not resolved in the cryo-EM map. **d** The N- and C-terminal intracellular domains are not required for hDisp1 activity in cells. WT hDisp1, the deletion mutants hDisp1^ΔN^ and hDisp1^ΔC^, or the inactive mutant hDisp1^NNN^, tagged as shown in the diagram, were stably co-expressed with Shh and HA-tagged Scube2 in Disp1-null HEK293T cells. The cells were incubated with serum-free media and Shh release after 24 h was assayed by SDS-PAGE and Western blotting. All constructs rescue Shh release, except the catalytically inactive mutant hDisp1^NNN^. Note that the low-percentage (5%) top portion of the gradient gel used to analyze the conditioned medium was not completely straightened, causing bending of the Scube2 bands on the blot. **e** Close-up showing the helix-swapped configuration of the two ECDs of hDisp1. Structure figures were prepared using UCSF Chimera^57^ or PyMol^58^. Source data for (**d**) are provided as a Source Data file.

To better understand the mechanism underlying cleavage-dependent activation of hDisp1, we set out to determine the structures of hDisp1^NNN^-3C before and after cleavage (referred to as hDisp1^NNN^-3C and hDisp1^NNN^-3C-cleaved), using single particle cryo-EM. We solved the structure of uncleaved hDisp1^NNN^-3C at overall resolution of 3.61 Å (Fig. 2a, b and Supplementary Fig. 2), and the structure of hDisp1^NNN^-3C-cleaved at 3.68 Å (Supplementary Figs. 3 and 4a, b). An N-terminal cytoplasmic segment consisting of 180 amino acid residues preceding TM1, and the C-terminal cytoplasmic segment consisting of 380 residues following TM12 were not resolved in either cryo-EM density map (Fig. 2c and Supplementary Fig. 4c), likely due to their intrinsic flexibility. However, these portions of the hDisp1 molecule are dispensable for function, as indicated by the fact that deletion mutants lacking either the N-terminal domain, or the C-terminal tail were able to efficiently rescue Scube-dependent Shh release in hDisp1-null cells (Fig. 2d). Most of the TMs and ECDs were well resolved in both the uncleaved and cleaved hDisp1 cryo-EM density maps, which permitted reliable model building with assignment of amino acid side chains (Supplementary Fig. 5). Four N-linked glycosylation sites were identified in both hDisp1 structures, Asn363 and Asn476 on ECD1, and Asn836 and Asn917 on ECD2, which, in turn, validated sequence assignment (Supplementary Fig. 6). Similar to the sterol-like molecules seen in the reported Ptch1 cryo-EM structures^23,26,28^, we observed several sterol-like molecules in the transmembrane domain (TMD) of both our hDisp1 structures; we assigned these molecules to the sterol detergent, cholesteryl hemisuccinate (CHS), which was used for protein purification (Supplementary Fig. 6).

Both hDisp1^NNN^-3C and hDisp1^NNN^-3C-cleaved display a fold typical of the RND family of transporters, with internal two-fold pseudosymmetry of the twelve TMs and the two ECDs, around an axis perpendicular to the membrane (Fig. 2a, b and Supplementary Fig. 4a, b). In contrast to other RND proteins, such as Ptch1^23,25,26^, in which the ECDs are close together, the two ECDs in hDisp1^NNN^-3C and hDisp1^NNN^-3C-cleaved are splayed apart, exhibiting an open conformation (Fig. 2a, b and Supplementary Fig. 4a, b), as previously reported for dDisp and hDisp1^32,33^. The two ECDs of hDisp1^NNN^-3C are held together through a helix-swapped configuration, with each ECD accepting secondary structure elements from the other ECD, involving helix α1 of ECD1 and helices α1 and α2 of ECD2 (Fig. 2e and Supplementary Fig. 7). Similar to Ptch1^36^, co-expressing the two halves of Disp1 as separate proteins reconstitutes activity in cells (Supplementary Fig. 4d), indicating that these non-covalent interactions suffice to assemble a functional Disp1 protein. A connecting loop between residues 263–285 in ECD1 (named the C-loop, for protease cleavage) is not well-resolved in our reconstruction, indicating its flexibility; we speculate that this permits its accessibility to Furin or 3C protease cleavage (Fig. 2b). While the absence of density for the C-loop creates the appearance of a large cavity between the two ECDs (Fig. 2a, b), it is important to emphasize that the C-loop, though unstructured, still occupies this space in uncleaved hDisp1^NNN^-3C (see below).

**Fig. 3:**
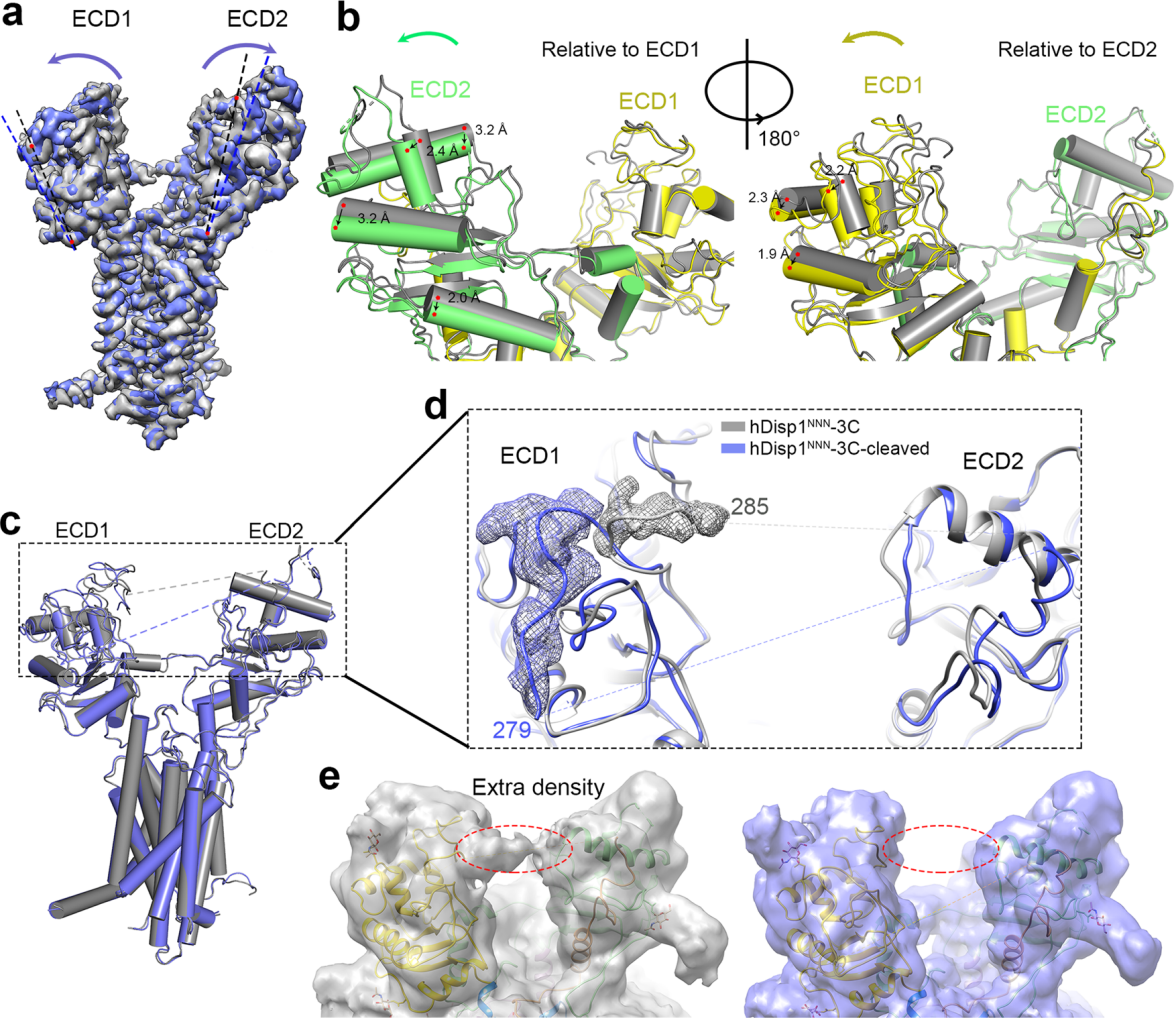
Conformational changes in hDisp1 induced by proteolytic cleavage. **a** Overall comparison of cryo-EM density maps for hDisp1^NNN^-3C, before (gray) and after cleavage (blue) by 3C protease. Proteolytic cleavage causes the two halves of the extracellular domain, ECD1 and ECD2, to move apart. **b** Two close-up views of the model for hDisp1, before (gray) and after cleavage (ECD1 yellow, ECD2 green). **c** As in (**a**), but showing hDisp1 models. **d** Close-up showing changes in the C-loop after 3C protease cleavage. Density maps before and after cleavage are represented as cyan and purple mesh, respectively. **e** Cryo-EM density map of uncleaved hDisp1 (left) shows extra density (red dotted oval), compared to cleaved hDisp1 (right).

A comparison between the structures of hDisp1^NNN^-3C and hDisp1^NNN^-3C-cleaved reveals conformational shifts of the two ECDs, whereas the TMDs remain largely unchanged (Fig. 3a–c). After proteolytic cleavage, the two ECDs turn outwardly, away from each other, by approximately 2–3 Å, leading to a more open conformation of the extracellular surface of hDisp1 (Fig. 3a, b). Additionally, density peaks for the C-loop in ECD1 were observed in the map of hDisp1^NNN^-3C-cleaved, enabling us to build 6 residues (residues 279–284) in the C-loop (Fig. 3d). The resolved C-loop is moved away from ECD2 in hDisp1^NNN^-3C-cleaved, suggesting that the uncleaved C-loop perhaps restricts the motions of the two ECDs, and that proteolytic cleavage allows them to move away from each other. Consistent with this model, extra density was observed at the C-loop position between the two ECDs in the EM map of uncleaved hDisp1^NNN^-3C at a low contour level, but no extra density was observed at the same position in the map of hDisp1^NNN^-3C-cleaved at the same or even lower contour level (Fig. 3e). Perhaps more importantly, cleavage of the C-loop, which occurs close to the site of connection with ECD1, permits the flexible loop to move outside of the region between the two ECDs, affording access to Shh (see below).

### A complex between hDisp1 and the native Shh ligand reveals requirements for Shh release

**Fig. 4:**
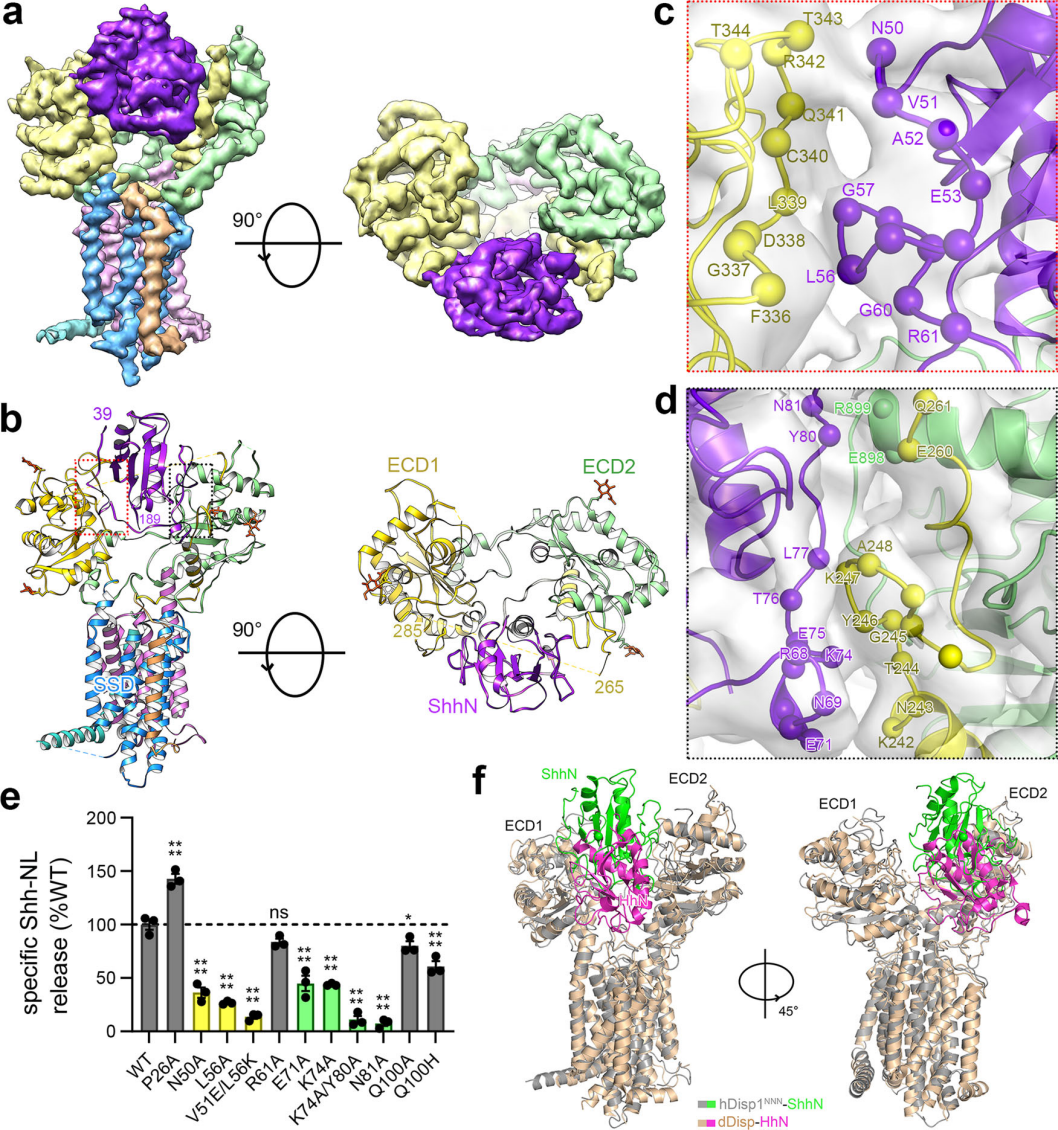
Structure of hDisp1 bound to native, dually lipidated Shh. **a**, **b** Corresponding views of the cryo-EM density map (**a**) and atomic model (**b**) of the hDisp1^NNN^–Shh complex. **c**, **d** The interaction interfaces between hDisp1 and Shh. Residues near the interfaces are shown as spheres. **e** WT and mutant Shh-NL constructs were transiently expressed in HEK293T cells together with WT Scube2 or the inactive Scube2 *ty97* mutant (negative control). Cells were washed extensively with serum-free media and Shh-NL release was measured after 6 h, for three independent biological replicates. Shh-NL release was normalized to Shh-NL measured in cell lysates, to account for differences in expression level, and specific Scube2-dependent release was determined by subtracting background Shh-NL release for Scube2 *ty97*. Bars represent mean background-subtracted Shh-NL release, and are plotted as percentage of release for WT Shh-NL. Error bars represent standard error of the mean. Ordinary one-way ANOVA, with Dunnett’s multiple comparisons test, was used to compare WT Shh-NL and each mutant: *, *p* < 0.05; ****, *p* < 0.0001; ns, not significant. Mutations are colored by their proximity to the Disp1–Shh interfaces, with yellow bars representing Shh residues close to ECD1 (N50A, V51E, L56A/K), green bars representing Shh residues close to ECD2 (E71A, K74A, Y80A, and N81A), and gray bars representing Shh residues at unrelated sites (P26A, R61A, Q100A, and Q100H). Mutations close to ECD1 and ECD2 show a significant defect (>2-fold reduction) in Shh release, while more distantly located mutations show modest or no reduction in Shh release. **f** Structural comparison between hDisp1–Shh and dDisp–Hh complexes shows that hDisp1 and dDisp have distinct ligand binding modes, consistent with divergent mechanisms of ligand release in vertebrates and invertebrates. Source data for (**e**) are provided as a Source Data file.

To further illuminate the mechanism of hDisp1-mediated Shh release, we generated a complex between hDisp1^NNN^ and the native, dually lipidated Shh ligand, and subjected it to cryo-EM analysis. After the application of an adapted mask on ECDs and Shh for focused 3D classification, we solved the structure of the complex at an overall resolution of 4.07 Å (Fig. 4a, b and Supplementary Fig. 8). The secondary structure features of Shh could be clearly resolved, allowing us to dock the crystal structure of Shh (PDB 4C4M) into the density map with manual adjustment.

A comparison between the structures of hDisp1^NNN^-3C-cleaved and that of the hDisp1^NNN^–ShhN complex reveals conformational shifts of the two ECDs, whereas no substantial conformational change in the TMDs is observed (Supplementary Fig. 9a). Upon Shh binding to hDisp1, Shh acts as a “molecular glue” that pulls the two ECDs of hDisp1^NNN^, turning them inwardly towards each other by approximately 4–5 Å (Supplementary Fig. 9b). In the hDisp1^NNN^–Shh complex structure, the two ECDs of hDisp1 grasp Shh like a pincer, with the N-terminus of Shh facing upwards and close to ECD1 and the C-terminus of Shh facing downwards and close to the SSD (Fig. 4b). Importantly, the Shh-binding site in hDisp1 clashes with the density corresponding to the uncleaved C-loop in the hDisp1^NNN^-3C structure (Supplementary Fig. 9c), which provides an explanation for the drastically reduced Shh binding that we observed for uncleaved hDisp1^NNN^-3C.

Although Shh was palmitate- and cholesterol-modified, we could not resolve the two lipid modifications, and thus any protein–lipid interactions between hDisp1 and Shh. However, our structure reveals an extensive protein–protein interaction between hDisp1 and Shh (Fig. 4a, b). This interaction involves two interfaces, one primarily between residues 336–343 of ECD1 and residues 50–58 of Shh (Fig. 4c), and the other primarily between residues 242–249/260–265 of ECD1 and residues 68–81 of Shh (Fig. 4d). Resolution of the cryo-EM map at the hDisp1–Shh interface is approximately 4.5 Å, precluding analysis of the specific interacting residues (Supplementary Fig. 8d). Nonetheless, several Shh variants bearing point mutations in ECD-adjacent residues exhibited reductions in their rate of Disp1- and Scube2-dependent release from cells, in contrast to Shh point mutations removed from the interface with hDisp1 (Fig. 4e and Supplementary Fig. 9d). To ensure that the Shh variants are properly trafficked to the cell surface, we performed immunofluorescence staining with or without detergent permeabilization (Supplementary Fig. 10). This analysis indicated that all but one of the mutants displayed cell surface localization similar to wild-type Shh, suggesting that the defects observed in release from cells are not due to impaired folding or trafficking. For the remaining mutant (Shh-N81A), surface localization was reduced just below 50%, suggesting that impaired trafficking, perhaps due to ER retention, is responsible, at least partially, for the observed release defect. Together, these results demonstrate that, in addition to the known requirement for lipid modifications in Disp1-mediated Shh release^13,14^, Shh recognition by hDisp1 via protein–protein interaction also plays a critical role in this process.

### Evolutionarily divergent aspects of Disp-catalyzed Hh ligand release

Dual lipidation of the Hh ligand and the essential role of Disp are conserved between vertebrates and invertebrates; however, the latter lack a Scube homolog, suggesting that another, yet unidentified, factor may be involved in a similar release mechanism. Consistent with this difference, the mode of Shh binding to hDisp1 that we observe is significantly different from *Drosophila* Hh binding to dDisp^32^, both in terms of ligand positioning relative to Disp and the ligand interaction interfaces (Fig. 4f); perhaps this explains why dDisp is only poorly able to promote Scube-mediated Shh release (Supplementary Fig. 9e). Further supporting evolutionary divergence between invertebrates and vertebrates, residues of Disp at the distinct ligand–Disp interfaces show a species-specific conservation pattern (Supplementary Fig. 11a, c). Together, these observations indicate that the mechanism of Hh release from producing cells is not strictly conserved across phyla.

## Discussion

**Fig. 5:**
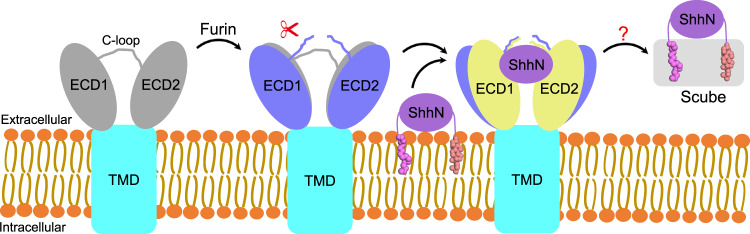
Model for hDisp1 activation by proteolytic cleavage. Disp1 is synthesized as an uncleaved precursor, in which the C-loop holds ECD1 and ECD2 together. In this conformation, Disp is inactive because it is sterically hindered from interacting with Shh. Proteolytic cleavage of the C-loop by Furin relieves the steric hindrance and opens the ECD, allowing the membrane-attached Shh to interact with Disp. The ligand is then handed off to the Scube protein, forming a soluble, signaling-competent Scube–Shh complex.

A longstanding question in Hh signaling has been how Hh morphogens spread to distant cells, in spite of dual lipidation with cholesterol and palmitate, which firmly anchors them to the surface of producing cells. In vertebrates, the Disp1 membrane transporter and the Scube family of secreted chaperones provide a biochemical solution to the problem of extracellular Shh release, with Disp1 extracting Shh from the membrane and catalyzing the formation of a soluble Scube–Shh complex^13–15^. However, Disp1 belongs to the RND family of small molecule transporters, and it has been unclear how Disp1 recognizes and transports a macromolecule such as Shh. Furthermore, Disp1 function requires a unique, conserved Furin-mediated proteolytic cleavage in its ECD, the role of which has also been unclear. Here, we use structural and functional experiments to address these open questions. By determining the structure of Disp1 before and after proteolytic cleavage, we discover a conformational change that opens the ECD and removes a steric impediment to Shh binding (Fig. 5); we thus speculate that proteolytic cleavage allows Disp1 to transport a protein, in contrast to all other RND protein family members which transport lipids and other small molecules. We also determine the structure of Disp1 bound to the native, dually lipidated Shh ligand, which reveals that the two halves of the Disp1 ECD move toward each other, to grasp Shh like a pincer, through two extensive protein–protein interfaces, which we demonstrate are critical for Shh release. Together, these results clarify how the Shh morphogen is secreted through the action of the Disp1 transporter, a critical step in the Hh pathway.

Genetic and biochemical evidence has suggested that Disp1 coordinates transfer of Shh lipid moieties to the Scube2 acceptor^12–15^, though the manner in which Scube2 interfaces with Disp1 and Shh remains unknown. Notably, Scube2 does not bind stably to wild-type Disp1, Disp1^NNN^, or Disp1-3C expressed in cells, whether or not Shh is present (Supplementary Fig. 12a). This observation suggests that Scube may transiently recognize an intermediate Disp1–Shh complex in the process of lipid extraction. While Scube was not included in our present structural studies, the orientation of Shh relative to Disp1 permits some speculation with regard to possible Scube–Shh interfaces. As positioned in the Disp1 pincer-like grasp, Shh presents two large solvent-exposed surfaces: a “front” surface, containing the pseudo-active site, which faces away from Disp1, and a “back” surface, accessible between the splayed ECD1 and ECD2 of Disp1 (Supplementary Fig. 12b). Conceivably, a Scube molecule could engage Shh lipids from either approach. However, the palmitoylated N-terminal peptide of Shh, which will be transferred to Scube^13,16^, projects from the “back” surface of Disp1-bound Shh. Additionally, insertion of NanoLuc luciferase into sites on the “front” surface of Shh does not impair Disp1- and Scube-dependent release^15^, while insertions into sites on the “back” surface of Shh abolish release (Supplementary Fig. 11b, inset)^15^. Finally, Shh can simultaneously bind Scube and the co-receptors Cdon/Boc^16^, the latter interaction involving the Shh pseudo-active site^37^. Together, these observations suggest that Scube likely engages Disp1-bound Shh from its “back” surface. Future structural studies of Scube–Shh, or of a potential ternary Disp1–Shh–Scube complex will be required to confirm this structure-guided speculation.

Previous work proposed that Disp1 transports the Shh cholesterol moiety from the membrane to Scube2^14,15^. As in previous Disp/Disp1 structures^32,33,38^, however, we were unable to resolve the lipid-modified termini of Shh, perhaps because their binding to Disp1 was disrupted by detergent present during protein purification. Nonetheless, our structural and functional studies of Furin-mediated proteolytic cleavage, together with structural studies of other eukaryotic RND transporters, permit some speculation with regard to the path of substrate transport through Disp1. The Disp1 homologs Ptch1^23,25,26^ and NPC1^39–41^ are proposed to transport cholesterol through a hydrophobic conduit comprised of the ECD1 and ECD2 interfaces (Supplementary Fig. 12c, d). Although ECD1 and ECD2 are splayed apart in Disp1 compared to Ptch1 and NPC1, it is thus tempting to speculate that the Shh cholesterol moiety is transported along a similar path through Disp1 (Supplementary Fig. 12e), particularly if Scube2 engages Shh from its “back” surface. Strikingly, in the absence of Furin cleavage, the C-loop of Disp1 would then pose a topological barrier to Shh release to Scube2, catching the C-terminal Shh peptide and entangling Shh with Disp1 (Supplementary Fig. 12f, g). While we directly show that Furin cleavage removes a steric impediment to the protein–protein interaction between Shh and Disp1, we speculate that cleavage may also open a path for the transfer of the Shh cholesterol moiety from Disp1 to Scube2 ((Supplementary Fig. 12h). It is noteworthy that both putative functions of Furin cleavage appear to be adaptations that allow Disp1 to transport a protein substrate, in a manner analogous to how small molecules are transported by other RND proteins.

The interaction between hDisp1 and Shh we observed is strikingly different from the interaction described between dDisp and the Hh ligand^32^. Invertebrates do not have Scube homologs, and it is currently unknown what factor fulfills its role; one candidate is the secreted Hh-binding protein Shifted (Shf)^42,43^. These observations indicate that Disp-mediated Hh release is significantly divergent between phyla, reminiscent of the distinct interaction modes seen in X-ray structures of homologous invertebrate Ihog–Hh and vertebrate Cdon–Shh complexes^37^. It is also noteworthy that the C-loop of dDisp, which is also cleaved^31^, is greatly expanded and contains predicted secondary structural elements (Supplementary Fig. 7), further consistent with divergent aspects of Hh release. A better understanding of the mechanism of Hh release in invertebrates will have to await the identification of the acceptor protein(s) to which Hh is transferred from Disp.

Aside from the interaction between Disp1 and the lipid moieties of Shh, structural studies show that Disp binding to the Hh ligand can be recapitulated in vitro with the unlipidated ligand, both in vertebrates^38^ and in invertebrates^32^. These results indicate that Disp recognizes the Hh ligand via both protein–lipid and protein–protein interactions, similar to how Ptch1 binds Shh^24,27^. An interesting aspect concerns how this dual interaction mode occurs in vertebrates versus invertebrates, given the lack of conservation of the protein–protein component. We speculate that the protein–lipid component of the Disp–ligand interaction is conserved across phyla, and a significant degree of flexibility allowed the emergence of distinct protein–protein interaction modes, adapted to the unrelated proteins employed in different phyla as Hh ligand acceptors.

## Methods

### Protein expression and purification

Constructs encoding WT hDisp1, hDisp1^NNN^ and hDisp1^NNN^-3C were generated in the pCAG vector, with an N-terminal Flag tag and a C-terminal His_10_ tag. HEK293F suspension cells were cultured in SMM 293T-II medium (Sino Biological Inc.) at 37 °C, under an atmosphere of 5% CO_2_. The cells were transiently transfected at a density of 2.0×10^6^ cells per mL, using polyethyleneimine (PEI) (Polysciences). For a one-liter cell culture, 1 mg plasmid DNA was mixed with 3 mg PEI in 50 mL fresh medium, for 15–30 min at room temperature (RT), after which the mixture was added to the suspension culture. After 12 h, the cell culture was supplemented with 10 mM sodium butyrate, to boost protein expression. The transfected cells were cultured for an additional 48 h, before harvesting.

For protein purification, the HEK293F cell pellet was resuspended in buffer containing 25 mM Tris pH 8.0, 150 mM NaCl, and protease inhibitor cocktails (Amresco). After sonication on ice, membranes were solubilized with 1% (w/v) DDM (Anatrace) and 0.2% CHS (Anatrace), for 2 h at 4 °C. After centrifugation at 20,000 × g for 1 h, the supernatant was applied to anti-Flag G1 affinity resin (GenScript). The resin was rinsed with wash buffer [25 mM Tris pH 8.0, 150 mM NaCl, and 0.02% GDN (w/v) (Anatrace)], and the protein was eluted with wash buffer supplemented with 0.2 mg/mL Flag peptide. The eluent was next applied to nickel affinity resin (Ni-NTA, Qiagen). After rinsing with wash buffer with 20 mM imidazole, the protein was eluted with wash buffer with 250 mM imidazole. The Ni-NTA eluent was concentrated and further purified by SEC (Superose 6 10/300 GL, GE Healthcare) in wash buffer. The fractions corresponding to the monomeric protein peak were pooled and concentrated to ~10 mg/mL for cryo-EM sample preparation.

To prepare the hDisp1^NNN^-3C-cleaved protein sample, purified hDisp1^NNN^-3C at ~10 mg/mL was mixed with 3C protease (1 μM), and was incubated overnight at 4 °C before cryo-EM sample preparation. To assemble the hDisp1^NNN^–ShhN complex, dually lipidated hShh (R&D Systems, cat. no. 8908-SH/CF) was mixed with ~10 mg/mL hDisp1^NNN^ (1.2:1 molar ratio), and was incubated for 2 h at 4 °C before cryo-EM sample preparation.

Mouse Scube2 (mScube2), tagged with one copy of the Flag epitope at the N-terminus, was expressed as secreted protein in *MGAT1* ^_^/− HEK293S cells, and was affinity purified from conditioned media, using beads coupled to anti-Flag-M1 antibody^15^.

### Western blotting

Following separation by SDS-PAGE, proteins were transferred onto Immobilon-P PVDF or nitrocellulose transfer membranes (Millipore). The membranes were blocked with 5% nonfat dry milk in TBST (Tris-buffered saline with 0.1% Tween 20), for 1 h at RT, followed by incubation with primary antibody, for 1 h at RT. The primary antibodies were mouse anti-Flag monoclonal antibody (Sangon Biotech), mouse anti-His monoclonal antibody (Sangon Biotech), mouse anti-Strep monoclonal antibody (IBA Lifesciences), rabbit anti-Shh monoclonal antibody (Cell Signaling Technology), mouse anti-tubulin monoclonal antibody (Sigma), rat anti-HA polyclonal antibody (Roche), mouse anti-human protein C (HPC) monoclonal antibody (A. C. Kruse, Harvard Medical School), or affinity purified rabbit anti-mCherry polyclonal antibodies^44^. Primary antibodies were used at a final concentration of 1 μg/mL, in blocking solution. After three 5-minute washes in TBST, the membranes were incubated with goat anti-mouse HRP-conjugated secondary antibody (Sangon Biotech), sheep anti-mouse HPR-conjugated secondary antibody (Jackson ImmunoResearch), or donkey anti-rabbit IgG–HRP conjugate (GE Healthcare). Secondary antibodies were used at a dilution of 1:5000, in blocking solution. Bound antibodies were visualized by chemiluminescence (UltraSignal hypersensitive ECL chemiluminescence substrate, 4A Biotech), on an Amersham Imager 600 (GE).

### Shh-hDisp1 pull-down assay

hDisp1, hDisp1-3C, hDisp1^NNN^, or hDisp1^NNN^-3C, tagged with an N-terminal Flag tag and a C-terminal Twin-Strep tag, was co-expressed with full-length hShh in HEK293F cells. A 500-mL cell culture was transiently transfected with 0.375 mg hDisp1 or hDisp1-3C plasmid, and 0.375 mg hShh plasmid. A 200-mL cell culture was transiently transfected with 0.15 mg hDisp1^NNN^ or hDisp1^NNN^-3C plasmid, and 0.15 mg hShh plasmid. After 12 h, the cell culture was supplemented with 10 mM sodium butyrate. After another 48 h, the cells were collected by centrifugation and were resuspended in buffer containing 25 mM Tris pH 8.0, 150 mM NaCl, and protease inhibitor cocktail. Membranes were solubilized with 1% (w/v) LMNG (Anatrace), for 2 h at 4 °C. After centrifugation at 20,000 × g for 1 h, the supernatant was applied to anti-Flag affinity resin. The resin was rinsed with wash buffer, and bound protein was eluted with wash buffer supplemented with 0.2 mg/mL Flag peptide. The eluent was then applied to Strep-Tactin resin (IBA Lifesciences). After rinsing with wash buffer, bound protein was eluted with BXT elution buffer (IBA Lifesciences) and was analyzed by Western blotting.

### Cell-based Shh release assays

For assaying Shh release kinetics by NanoLuc (NL) luciferase assay, human Shh constructs (wild type and mutants) were tagged with NL, which was inserted between residues N91 and T92^15^. Shh-NL constructs were stably expressed in wild-type or hDisp1-null HEK293T cells^15^ by lentiviral transduction. For assaying Shh release at a fixed time by immunoblot, wild-type Shh was stably expressed in wild-type HEK293T cells. Where indicated, cells were also transduced with: wild-type hDisp1 (tagged with mCherry at the C-terminus); hDisp1^NNN^ (triple mutant D572N, D573N, D1051N, tagged with mCherry at the C-terminus); hDisp1-3C (tagged with the HPC epitope at the N-terminus and mCherry at the C-terminus); hDisp1 lacking residues M1 to F175, corresponding to the N-terminal intracellular domain (tagged with mCherry at the C-terminus); hDisp1 lacking residues G1141 to L1524, corresponding to the C-terminal intracellular domain; residues M1 to Q674 of hDisp1, corresponding to the first half of the protein (tagged with the HPC epitope at the N-terminus); residues Q676 to L1524 of hDisp1, corresponding to the second half of the protein (tagged with the HA epitope at the C-terminus); or dDisp (tagged with mCherry at the C-terminus). Cells were washed extensively with DMEM, to remove serum, and were preincubated with cycloheximide (100 μg/mL) for 30 min, to block new Shh-NL synthesis. Cells were then incubated with 1 µM purified Scube2 or BSA (negative control), and NL luciferase activity released into the media was measured at 4-min intervals for a total of ~20 min, using a Wallac VICTOR3 microplate reader and associated acquisition software (Perkin-Elmer). Activity released by BSA was subtracted from activity released by Scube2, to calculate specific activity released at each timepoint, and initial release rates were calculated by linear regression. To test the role of hDisp1-3C cleavage in Shh release, cells were treated or not with purified 3C protease (2 µM), prior to Scube2 addition. To compare release rates of Shh point mutants, released NL activity was normalized to total NL activity in lysates of Shh-NL-expressing stable cell lines, to account for any differences in Shh-NL expression level. Release rates were further normalized to wild-type Shh release rate (set to 100%). In experiments in which Shh-NL release was measured after 6 h, the indicated components were expressed by transfection, and cycloheximide was omitted. Cycloheximide was also omitted from Shh release experiments in which media and cells were collected after 24 h and were analyzed by immunoblotting. Analysis of covariance (ANCOVA) was performed in Prism to assess whether apparent differences in the best-fit slopes of the release timecourse data were statistically significant. One-way ANOVA was performed in Prism to assess the statistical significance of differences measured in 6-h endpoint release assays, which were performed with three biological replicates. Where necessary, reported p-values are adjusted for multiple comparisons, as indicated in the figure legends.

### Immunofluorescence

HEK293T cells expressing various Shh-NL mutants were grown on poly-lysine-coated glass coverslips and were fixed in PBS with 2% methanol-free formaldehyde (Thermo), for 30 min at room temperature. For cell surface staining (no permeabilization), all subsequent incubations were performed in the absence of detergent, while for total cell staining (with permeabilization), all incubations included 0.2% Triton-X100. Mouse monoclonal antibody against NanoLuc luciferase (Promega) was used at a final concentration of 1 μg/mL in TBS or TBST with 3% bovine serum albumin (BSA). The secondary antibody, goat anti-mouse IgG–Alexa Fluor 594 conjugate (Thermo), was used at 1 μg/mL in TBS or TBST with 3% bovine serum albumin (BSA). After staining, the coverslips were mounted in PBS with 50% glycerol and were imaged on a Nikon TE2000E epifluorescence microscope, equipped with an OrcaER camera (Hamamatsu) and 20x PlanApo 0.75NA air objective (Nikon). Images were acquired using MetaMorph software (Molecular Devices), for two different fields of view per condition. For each fluorescence image, the corresponding transmitted light image was acquired by DIC. Condition-blinded manual segmentation in Fiji was used to quantify total and surface Shh-NL staining. For each condition, the average background-subtracted fluorescence intensity was measured for 40 circular regions of interest (20-pixel diameter), manually drawn over regions of the image containing cells, as assessed via the DIC channel image. Background fluorescence was provided by staining in parallel HEK293T cells expressing an unrelated secreted protein (HaloTag). Staining for each Shh-NL variant is reported as the average of the 40 regions, with error bars representing SEM. The montage of representative images was assembled in MetaMorph.

### Cryo-EM sample preparation and data collection

Aliquots (3.5 µL) of hDisp1^NNN^-3C, hDisp1^NNN^-3C-cleaved, and the hDisp1^NNN^–ShhN complex were applied to glow-discharged grids (Quantifoil, R1.2/1.3 Cu, 300 mesh), which were then blotted for 3.5 s and plunged into liquid ethane using a Vitrobot (Mark IV, Thermo Fisher FEI), operated at 8 °C and 100% humidity.

For the hDisp1^NNN^-3C and hDisp1^NNN^-3C-cleaved data sets, micrographs were collected at 300 kV on a Titan Krios microscope (#1 Titan Krios) equipped with a K2 Summit direct electron detector (Gatan), using a slit width of 20 eV on a GIF Quantum energy filter (Gatan). SerialEM^45^ software was used for automated data collection under super-resolution mode with a nominal magnification of × 130,000, yielding a pixel size of 1.08 Å. The defocus range was set from −2.0 to −1.0 µm. Each micrograph was dose-fractionated to 32 frames under a dose rate of 9.6 e^-^/pixel/s, with a total exposure time of 5.76 s, resulting in a total dose of approximately 50 e^-^/Å2. For the hDisp1^NNN^–ShhN complex data set, micrographs were collected in a similar manner on another Titan Krios microscope (#2 Titan Krios) with a configuration similar to that of #1 Titan Krios, at a nominal magnification of × 105,000 with defocus values from −2.0 to −1.2 μm, yielding a pixel size of 1.114 Å.

### Image processing

Motion correction and dose-weighted motion correction were performed using MotionCor2^46^. Gctf^47^ was used for CTF parameter estimation. For the hDisp1^NNN^-3C and hDisp1^NNN^-3C-cleaved data sets, particles were automatically picked and extracted in Relion 3.0^48^, and were subjected to several rounds of 2D classifications. The 2,341,462 and 542,472 selected particles from the last 2D classification were subjected to two rounds of 3D classification. The good classes were selected and re-extracted for a round of autorefinement. Following that, 1,126,766 and 222,287 particles were subjected to a third round of 3D classification without alignment, after which a total of 159,333 and 63,043 particles were selected for further refinement and postprocessing, yielding final reconstructions at overall resolutions of 3.61 Å and 3.68 Å, respectively.

Data processing for the hDisp1^NNN^–ShhN complex was similar to that described above. Particles were automatically picked in Relion 3.0, and several rounds of 2D classification resulted in 2,794,419 good particles, which were subjected to two subsequent rounds of 3D classification. Then, 685,796 particles were selected for a round of autorefinement. To improve ShhN density, the selected particles after refinement were subjected to a third round of focused 3D classification with a soft mask covering the ECDs and ShhN. The best class containing 293,391 particles was selected and subjected to another round of focused 3D classification with an adapted soft mask. Finally, 173,169 good particles were selected for refinement and postprocessing, yielding a final density map at an overall resolution of 4.07 Å.

All 2D classification, 3D classification, and 3D autorefinement procedures were performed using Relion 3.0. Resolutions were estimated using the gold-standard Fourier shell correlation (FSC) 0.143 criterion^49^, with high-resolution noise substitution^50^.

### Model building and refinement

The de novo atomic models of hDisp1^NNN^-3C and hDisp1^NNN^-3C-cleaved were built in Coot^51^, guided mainly by bulky residues such as Phe, Tyr, Trp and Arg. Each residue was manually checked, and its chemical properties were taken into account during model building. For the hDisp1^NNN^–ShhN complex, the crystal structure of ShhN (PDB 4C4M) and the structural model of hDisp1^NNN^-3C-cleaved were fitted into the density map, followed by manual adjustment. The structural models were refined using Phenix^52^ in real space with secondary structure and geometry restraints. The final refinement statistics are summarized in Table S1.

### Sequence conservation analysis

Sequence alignments were performed using Clustal Omega server^53^ and ESPript server^54^ and the conservation analysis was performed using the ConSurf Server^55^.

### Cell-based Scube2 binding assay

Binding of purified Scube2 to mCherry-tagged membrane proteins was performed in HEK293T cells^15^. The cells were plated in 48-well plates coated with poly-d-lysine, and were then transiently transfected with mCherry-tagged constructs. Two days after transfection, cells were incubated with purified Flag-tagged Scube2 in DMEM at a final concentration of 1 µM, for 1.5 h at 37 °C. Cells were washed once with DMEM, fixed with 3.7% (m/v) formaldehyde in PBS, treated for 3 min with methanol at −20 °C, and then stained with AlexaFluor488-labeled anti-Flag-M1 antibodies. Images were collected on a Nikon TE2000-E inverted microscope controlled by MetaMorph software, using a 10x PlanApo 0.45NA air objective (Nikon), followed by image analysis in MATLAB^15^. Briefly, mCherry-positive cells were segmented, and the corresponding background-subtracted anti-Flag fluorescence intensity was calculated for each cell object. The distribution of ratios of Scube2 intensity to area for mCherry-positive cells is represented as boxplots, as described in the figure legend.

### Statistics and reproducibility

Unless otherwise indicated in the figure legends, the results presented in all the SDS-PAGE, Western blotting and cryo-EM micrographs are representative of at least three independent experiments. Numerical data were analyzed in MATLAB, Microsoft Excel, and Prism.

### Reporting summary

Further information on research design is available in the Nature Research Reporting Summary linked to this article.

## Supplementary information

Supplementray Information

Reporting Summary

## Data Availability

The cryo-EM maps of hDisp1^NNN^-3C, hDisp1^NNN^-3C-cleaved and hDisp1^NNN^–ShhN have been deposited in the Electron Microscopy Data Bank (EMDB) with accession codes EMD-30956, EMD-30957 and EMD-30958, respectively. The corresponding atomic coordinates have been deposited in the Protein Data Bank (PDB) with accession codes 7E2G [10.2210/pdb7E2G/pdb], 7E2H [10.2210/pdb7E2H/pdb] and 7E2I [10.2210/pdb7E2I/pdb], respectively. Source data are provided with this paper.

## Source data

Source Data
