# Supplementary material for: Structural insights into proteolytic activation of the human Dispatched1 transporter for Hedgehog morphogen release
Source: Nat Commun. 2021 Nov 29;12:6966. doi: 10.1038/s41467-021-27257-w (PMC8630017; doi:10.1038/s41467-021-27257-w)
Supplement: Supplementary file 1 — Supplementray Information [file 41467_2021_27257_MOESM1_ESM.pdf]

Supplementary Information

Structural insights into proteolytic activation of the human Dispatched1 transporter for Hedgehog morphogen release

Wanqiu Li[1,4,6], Linlin Wang[1,4], Bradley M Wierbowski[2,4], Mo Lu[1], Feitong, Dong[1], Wenchen Liu[1], Sisi Li[1,7], Peiyi Wang[3], Adrian Salic[2,5], and Xin Gong[1,5]

[1]Department of Biology, School of Life Sciences, Southern University of Science and Technology, Shenzhen 518055, Guangdong, China

[2]Department of Cell Biology, Harvard Medical School, Boston MA 02115, USA

[3]SUSTech Cryo-EM Facility Center, Southern University of Science and Technology, Shenzhen 518055, Guangdong, China

[4]These authors contributed equally to this work.

[5]To whom correspondence should be addressed: A. Salic (asalic@hms.harvard.edu) or X. Gong (gongx@sustech.edu.cn).

[6]Present address: Department of Pharmacology, School of Medicine, Southern University of Science and Technology, Shenzhen 518055, Guangdong, China

[7]Present address: Department of Biochemistry and Molecular Biology, International Cancer Center, Shenzhen University Health Science Center, Shenzhen 518060, Guangdong, China

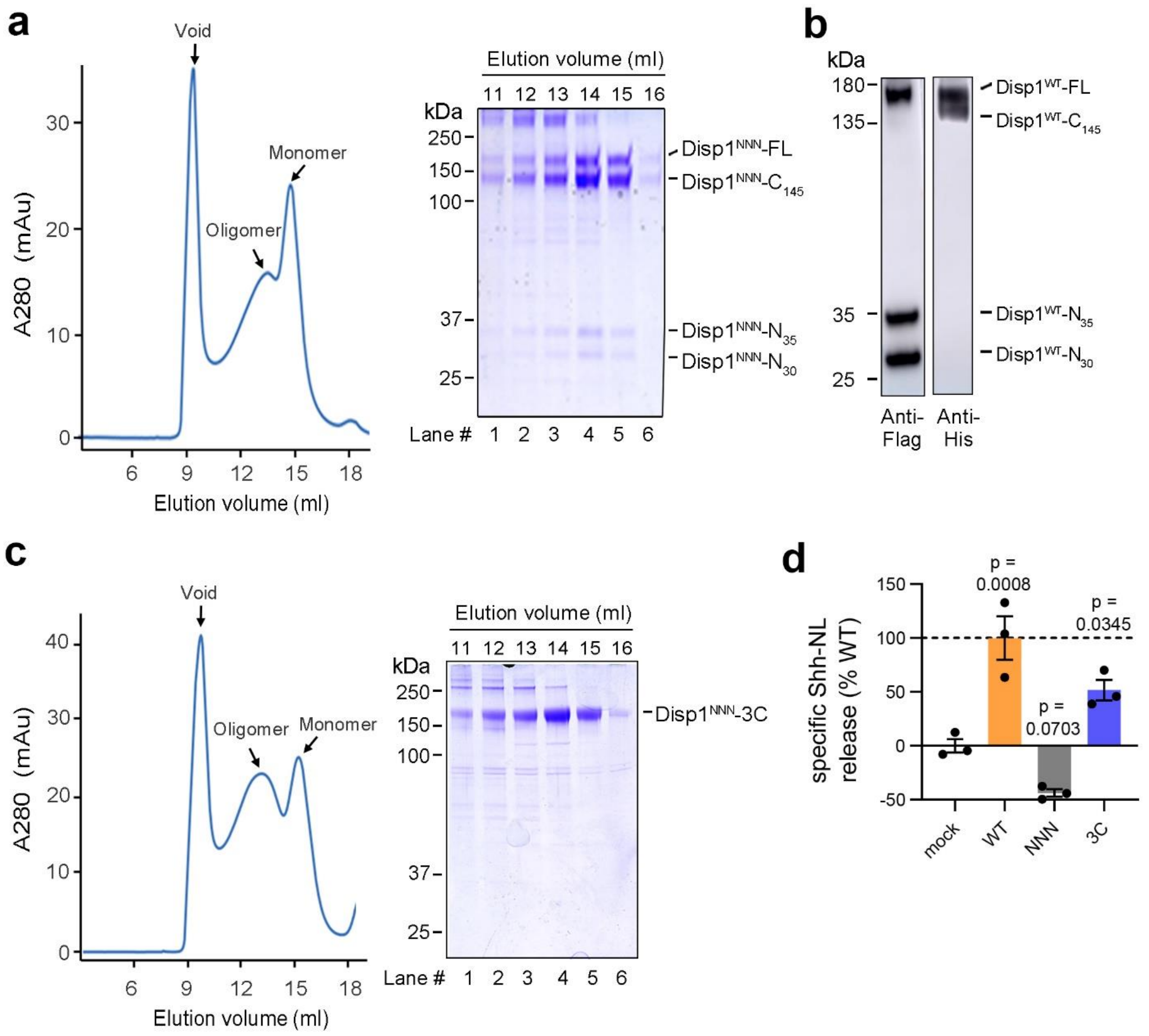

Supplementary Fig. 1 | Purification of hDisp1^{NNN} and hDisp1^{NNN}-3C proteins, and proteolytic processing of hDisp1 and hDisp1-3C. **a**, Size exclusion chromatography (SEC) profile and Coomassie blue-stained SDS-PAGE gel corresponding to the last step in the purification of the hDisp1 mutant, hDisp1^{NNN}. Fractions corresponding to monomeric species were pooled and concentrated, and were subjected to cryo-EM analysis. **b**, Purified wild type (WT) hDisp1 was separated by SDS-PAGE, followed by Western blot detection. Full length and cleavage products are indicated. Purified WT hDisp1 consists of a mix of cleaved and uncleaved species, similar to hDisp1^{NNN}. **c**, Size exclusion chromatography (SEC) profile and Coomassie blue-stained SDS-

PAGE gel corresponding to the last step in the purification of hDisp1^{NNN}-3C. **d,** WT hDisp1, the inactive hDisp1-NNN mutant, or hDisp1-3C were transiently co-expressed with wild-type Scube2 or the inactive mutant Scube2 *ty97* in Disp1-null HEK293T cells stably expressing Nanoluciferase-tagged Shh (Shh-NL). Cells were washed extensively with serum-free media and Shh-NL release was measured after a 6-hour incubation with 2μM 3C protease in three independent biological experiments. Released Shh-NL was normalized to Shh-NL measured in cell lysates, to account for differences in expression. Specific Scube2-dependent Shh-NL release was determined by subtracting background release by Scube2 *ty97*. Bars represent mean specific Shh-NL release, normalized between release from mock-transfected cells (0%) and release from cells transfected with WT hDisp1 (100%). Error bars represent standard error of the mean. Ordinary one-way ANOVA, with Dunnett's multiple comparisons test, was used to compare mock-transfected cells and each hDisp1 variant. hDisp1-3C cleaved with 3C protease is active in releasing Shh from cells, similar to WT Disp1. Note that cells overexpressing hDisp1-NNN release Shh slower than mock-transfected cells, perhaps due to Shh sequestration by hDisp1-NNN (ref 14, Tukachinsky et al., 2012). Source data for **a**-**d** are provided as a Source Data file.

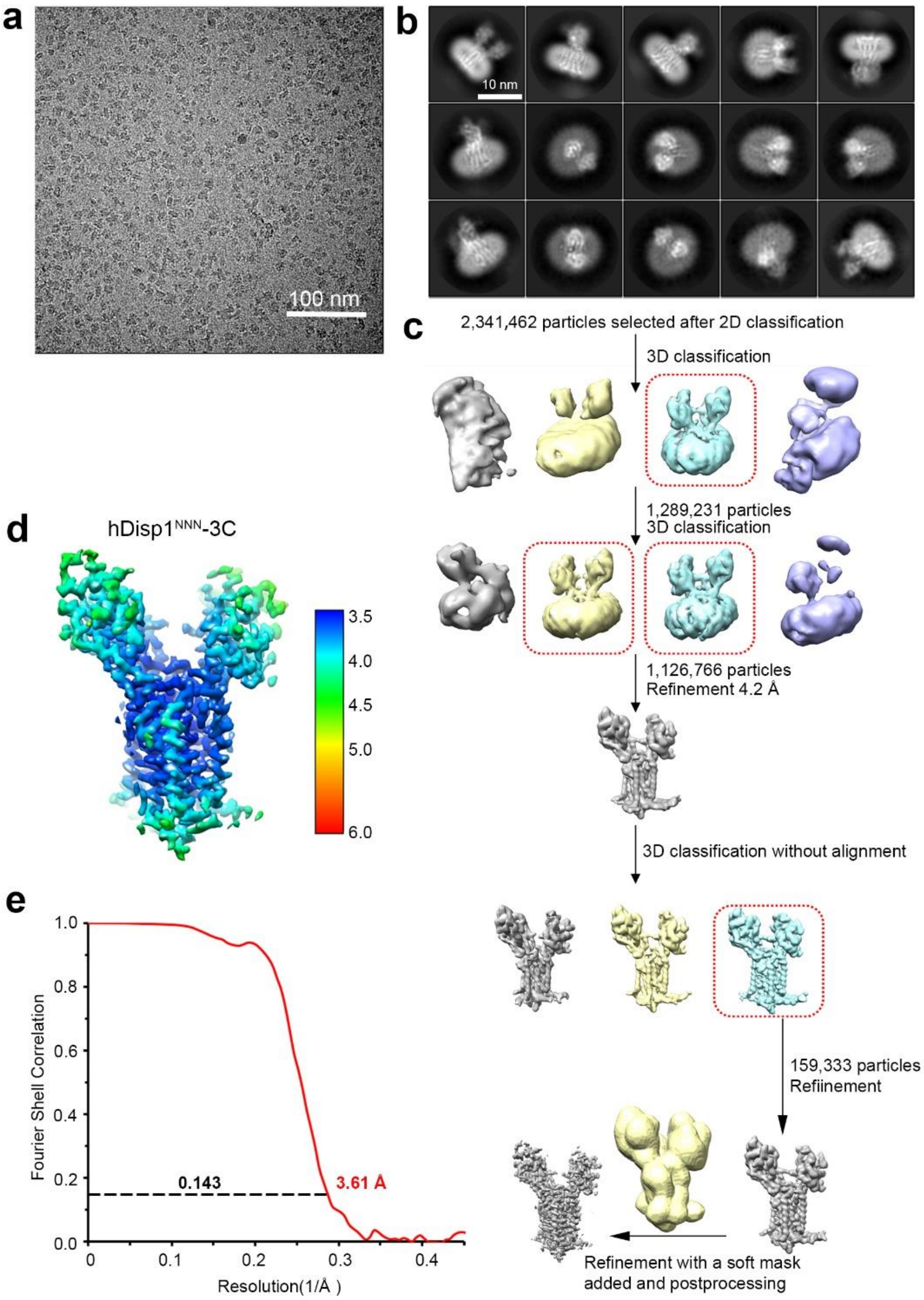

Supplementary Fig. 2 | Cryo-EM analysis of uncleaved hDisp1 (hDisp1^{NNN}-3C). **a**, A representative motion-corrected cryo-EM micrograph. **b**, Representative 2D class averages. **c**, Overview of the image processing workflow. **d**, Local resolution map calculated using Relion 3.0. **e**, Gold-standard Fourier shell correlation (FSC) curve for the refined map.

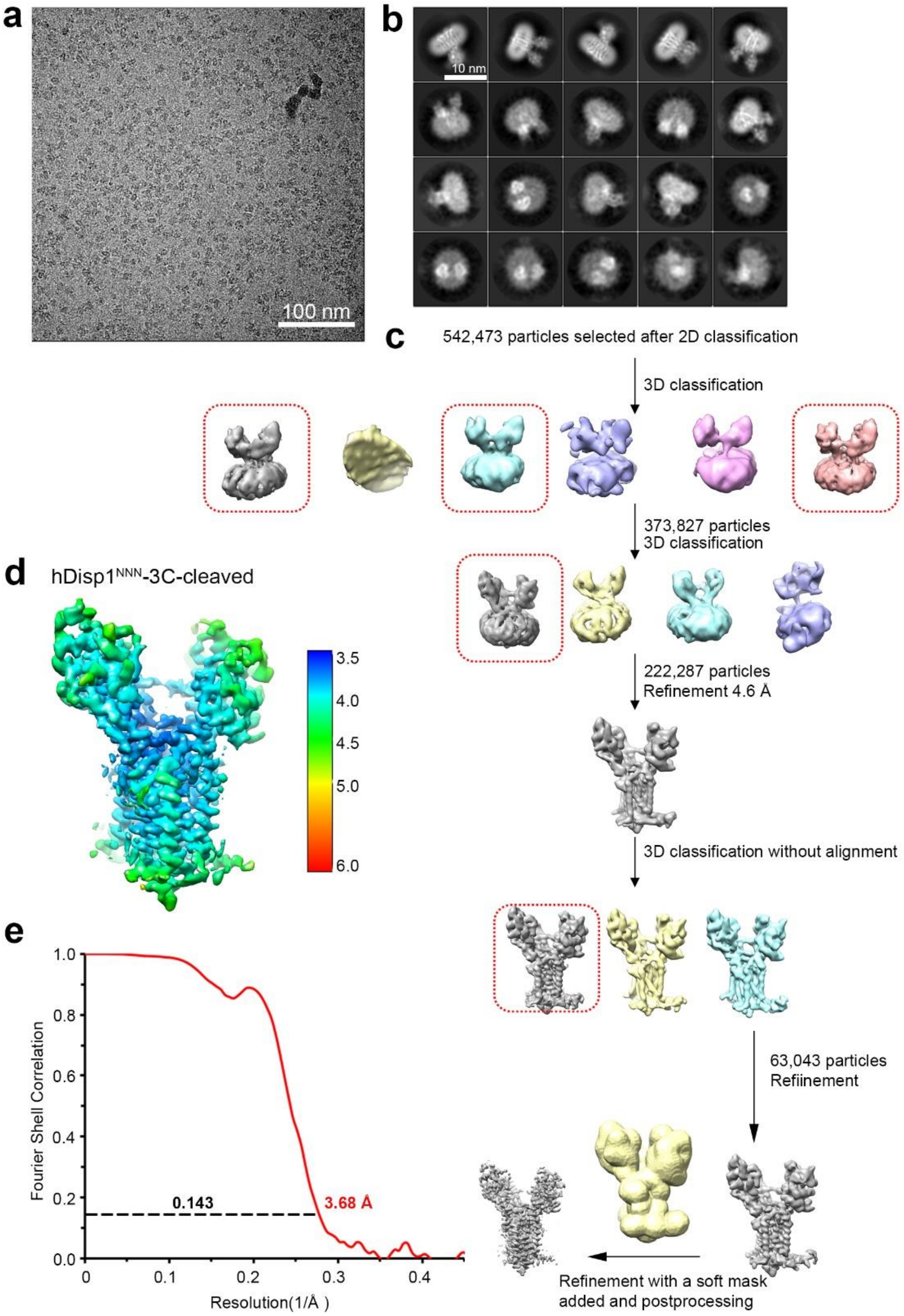

Supplementary Fig. 3 | Cryo-EM analysis of cleaved hDisp1 (hDisp1^{NNN}-3C-cleaved). **a**, A representative motion-corrected cryo-EM micrograph. **b**, Representative 2D class averages. **c**, Overview of the image processing workflow. **d**, Local resolution map calculated using Relion 3.0. **e**, Gold-standard FSC curve for the refined map.

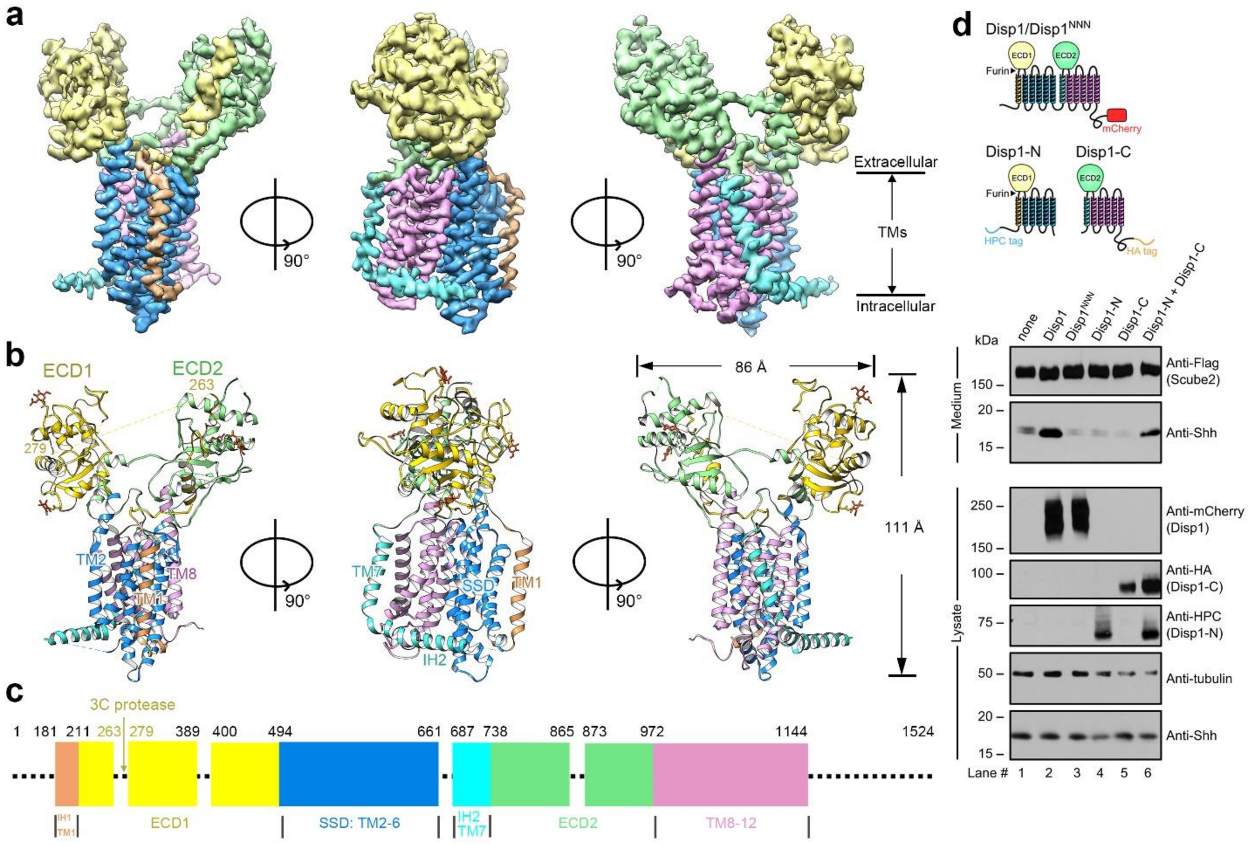

Supplementary Fig. 4 | Overall structure of cleaved hDisp1 (hDisp1^{NNN}-3C-cleaved). **a**, **b**, Corresponding views of the cryo-EM density map (**a**) and atomic model (**b**) of hDisp1^{NNN}-3C-cleaved. Domains are colored as in Fig. 2. **c**, Sequence coverage of the atomic model and annotation of the sequence. Dashed lines indicate the portions of the protein that were not resolved in the cryo-EM map. **d**, Constructs encoding the two halves of hDisp1 (hDisp1-N and hDisp1-C), tagged as in the diagram above, were co-expressed with Shh in Disp1-null HEK293T cells. The cells were incubated with 1 μM purified Scube2 in serum-free media, and Shh release after 24 hours was assayed by Western blotting. When expressed together, hDisp1-N and hDisp1-C reconstitute activity, which each half of hDisp1 expressed alone is inactive. WT hDisp1 served as positive control, and the inactive mutant hDisp1^{NNN} as negative control. Source data for **d** are provided as a Source Data file.

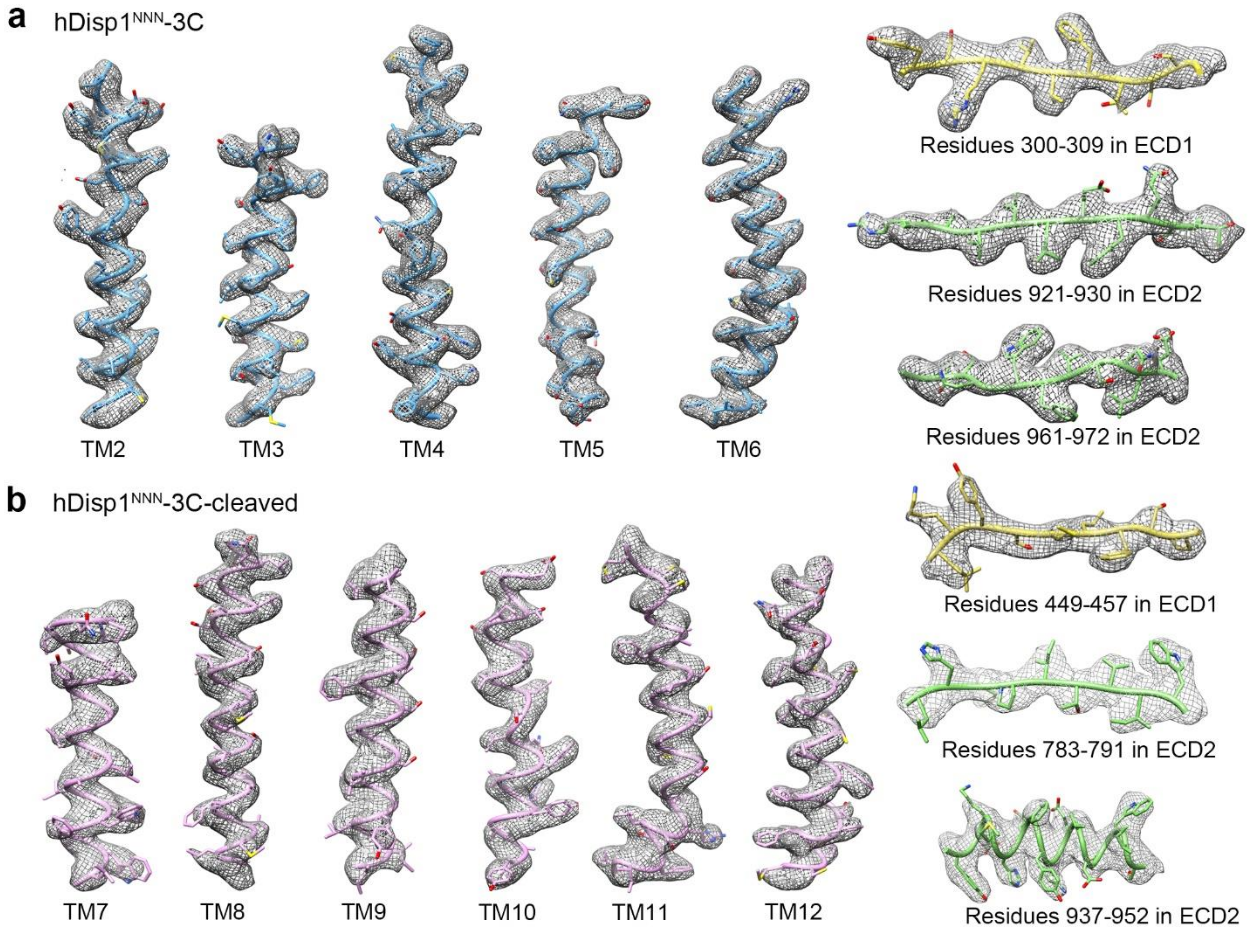

Supplementary Fig. 5 | Cryo-EM maps of representative segments in uncleaved (a) and cleaved (b) hDisp1.

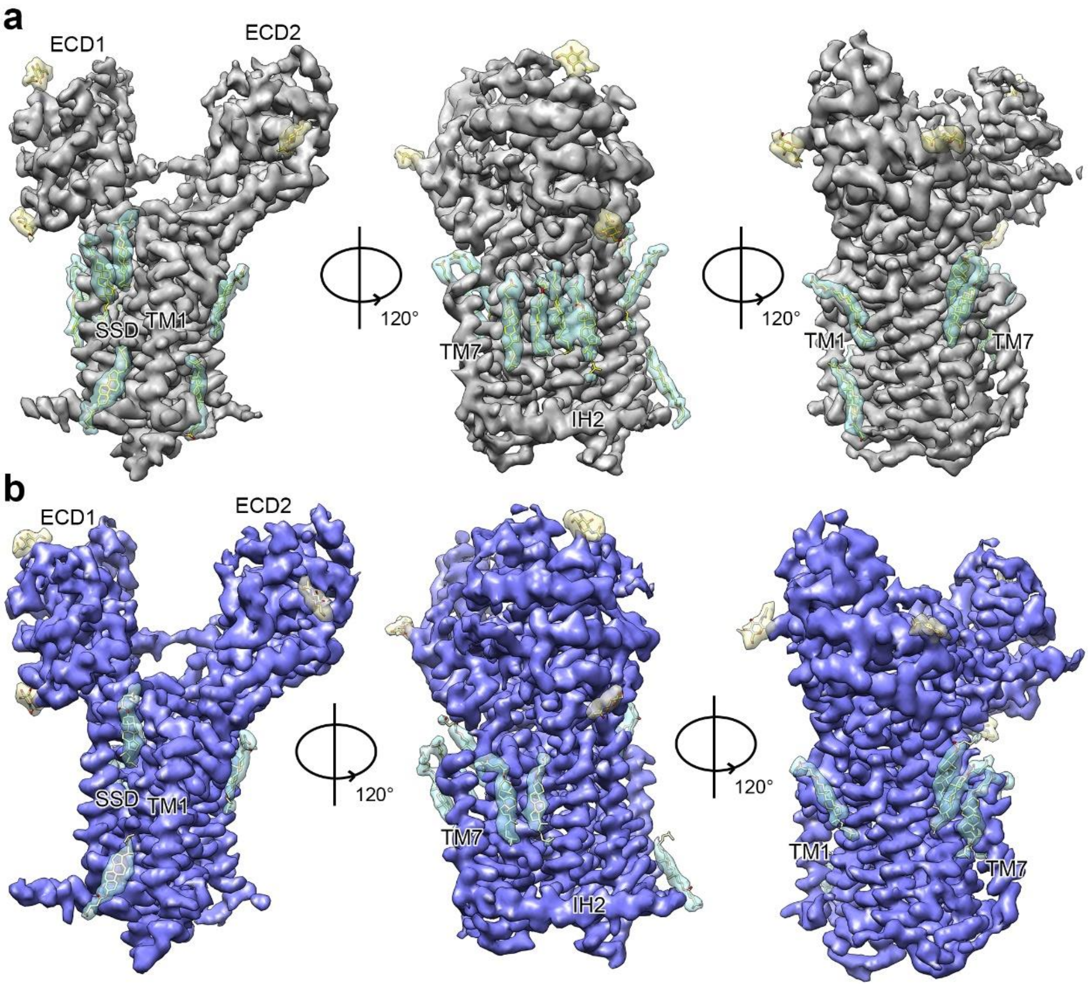

Supplementary Fig. 6 | N-linked glycosylation sites on the extracellular domain and sterol-like molecules in the TM region of the 3D reconstructions of hDisp1^{NNN}-3C (a) and hDisp1^{NNN}-3C-cleaved (b). Density for N-linked glycosylation sites and sterol-like molecules are shown as transparent yellow and green surface, respectively. N-acetyl-D-glucosamine (NAG) and cholesteryl hemisuccinate (CHS) molecules were modeled into the density for N-linked glycosylation sites and sterol-like molecules, respectively.

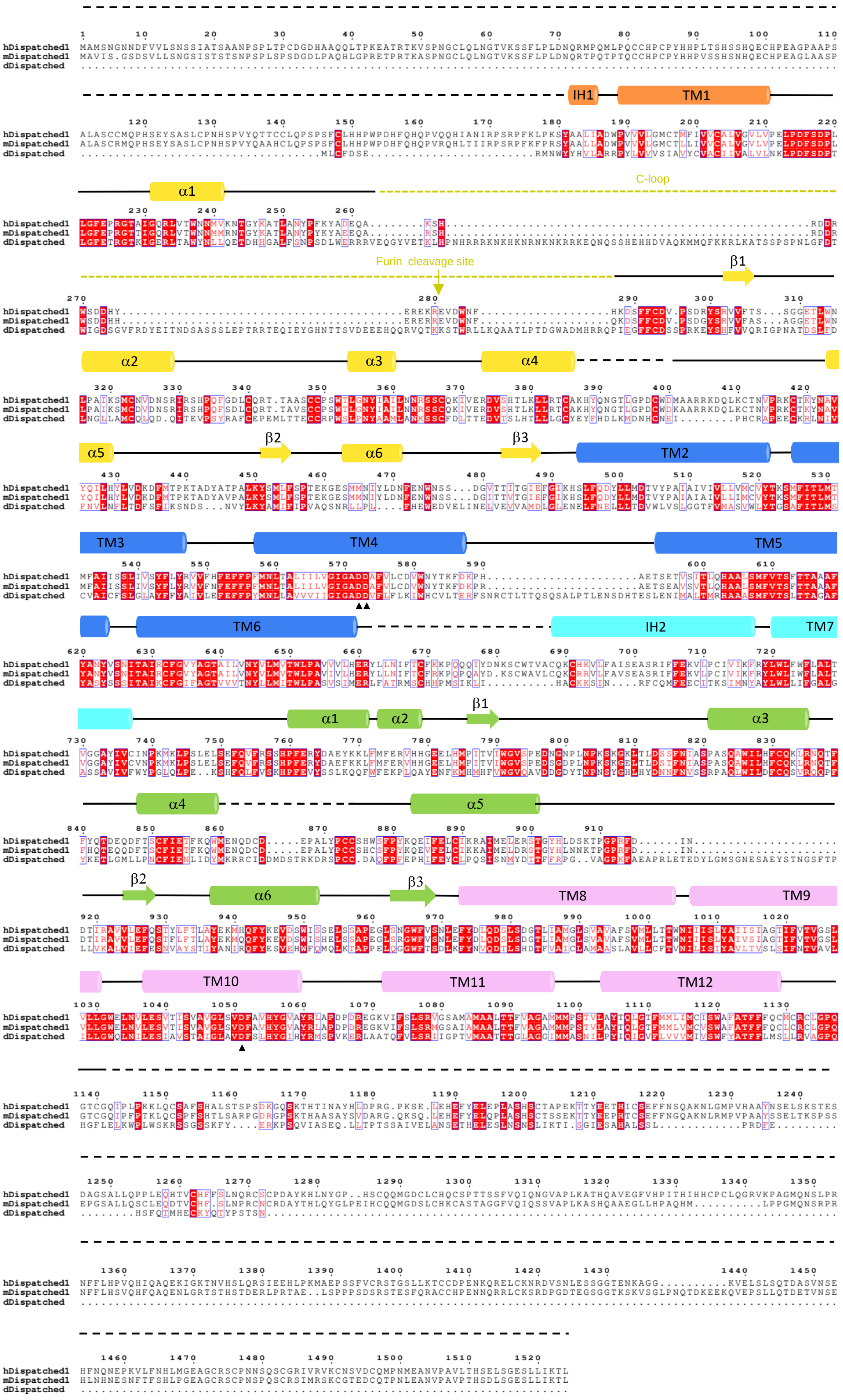
hDispatched1
mDispatched1
dDispatched
IH1
TM1
α1
C-loop
Furin cleavage site
β1
α2
α3
α4
α5
β2
α6
β3
TM2
TM3
TM4
TM5
TM6
IH2
TM7
α1
α2
β1
α3
α4
α5
β2
α6
β3
TM8
TM9
TM10
TM11
TM12

Supplementary Fig. 7 | Sequence alignment of human Disp1, mouse Disp1 and *Drosophila* Disp. Secondary structural elements of hDisp1^{NNN}-3C are indicated above the sequence alignment and color-coded using the same scheme described in Fig. 2. Dashed lines indicate protein portions that were not resolved in the cryo-EM map. The C-loop, which is responsible for Furin cleavage, is highlighted with a yellow dashed line. Note the massive expansion of the C-loop in dDisp, consistent with the involvement of distinct ligand acceptors downstream of Disp, in invertebrates versus vertebrates (see Discussion). The yellow arrow indicates the Furin cleavage site. The three conserved aspartate residues in TM4 and TM10 (Asp572, Asp573, and Asp1051) that were mutated to asparagine residues in hDisp1^{NNN} are highlighted by black triangles.

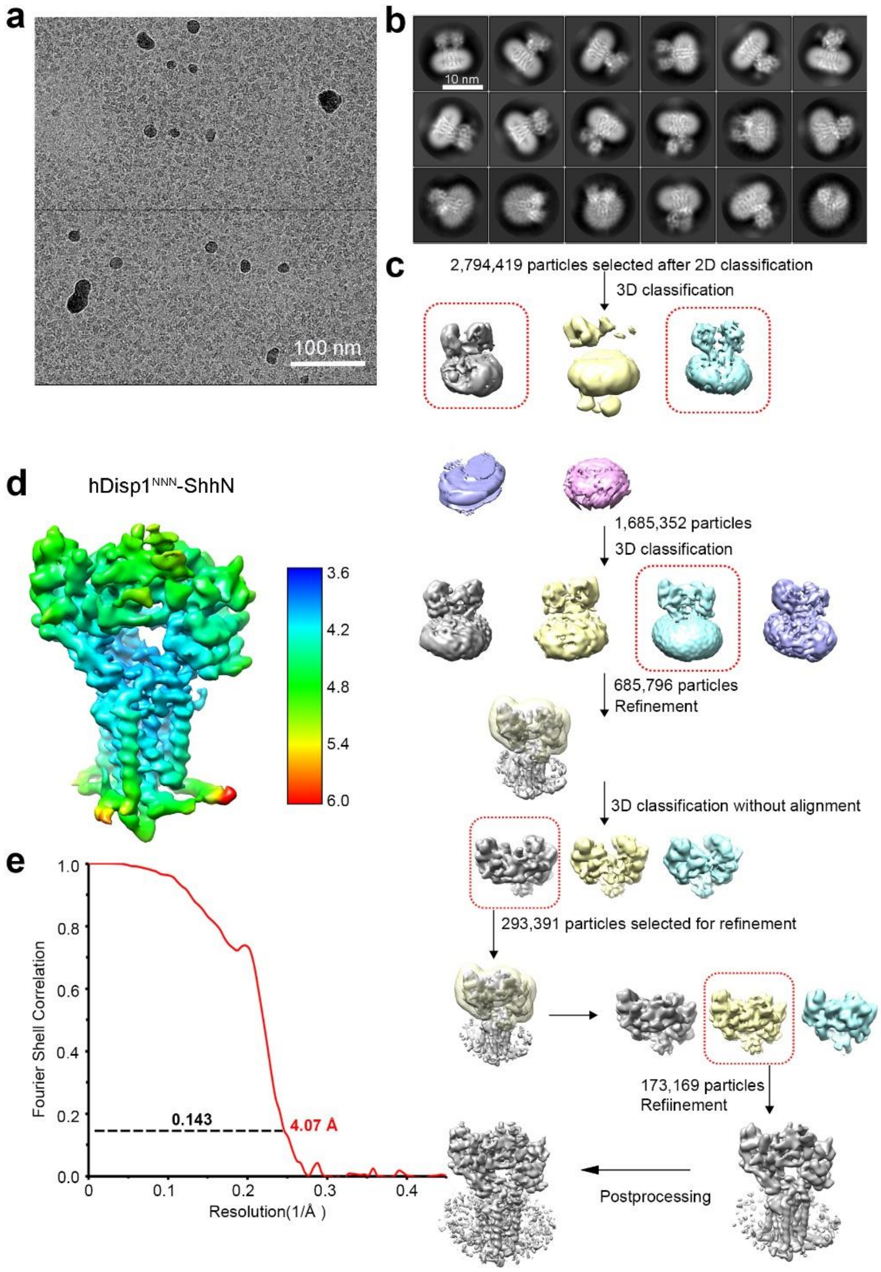

Supplementary Fig. 8 | Cryo-EM analysis of the hDisp1^{NNN}-Shh complex. The complex was assembled by mixing hDisp1^{NNN} and dually lipidated human Shh at a 1:1.2 molar ratio, and was then subjected to single particle cryo-EM structure determination. **a**, A representative motion-corrected cryo-EM micrograph. **b**, Representative 2D class averages. **c**, Overview of the image processing workflow. **d**, Local resolution map calculated using Relion 3.0. **e**, Gold-standard FSC curve for the refined map.

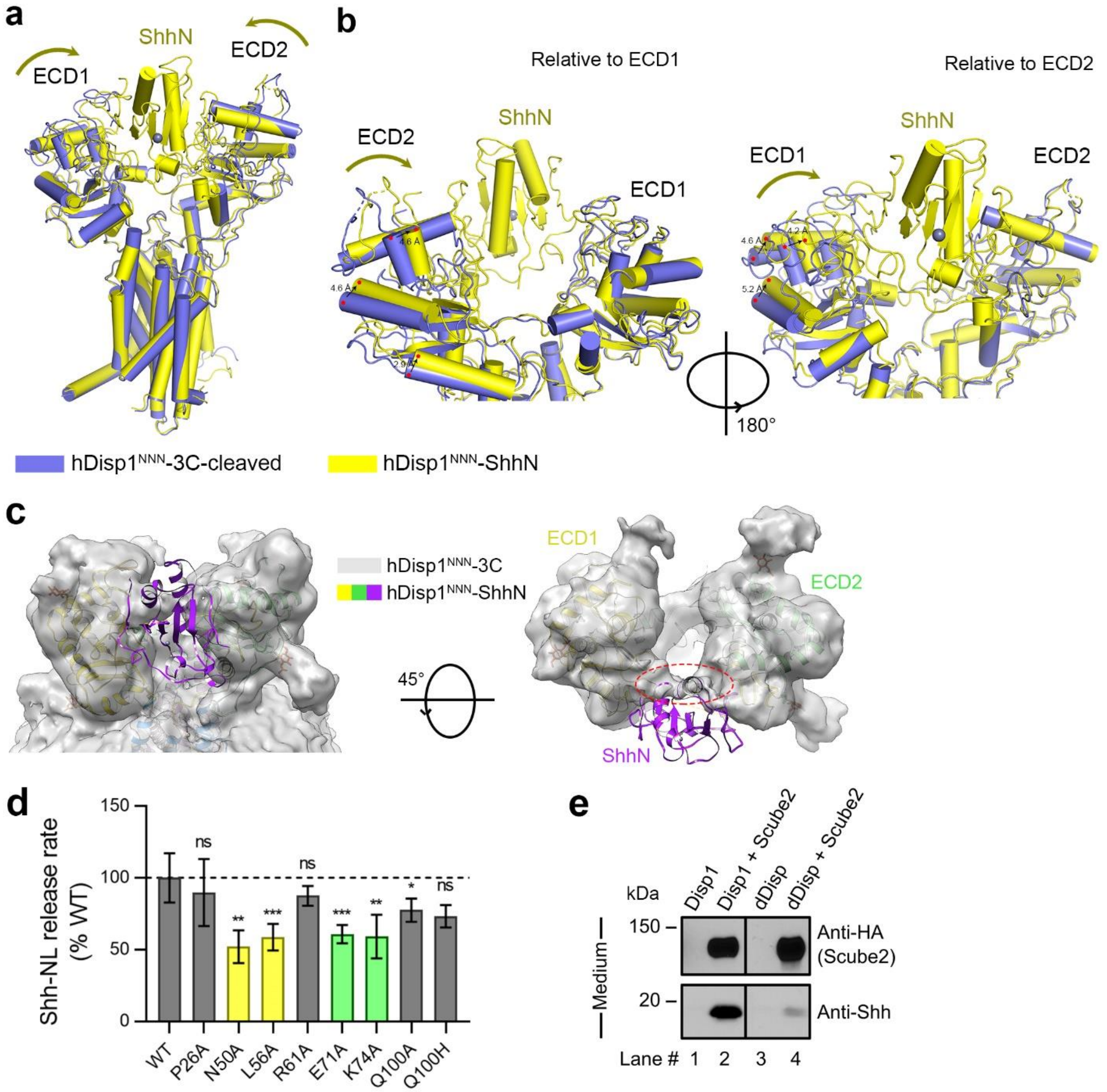

Supplementary Fig. 9 | Structural analysis of the hDisp1^{NNN}-ShhN complex. **a**, **b**, Comparison between the hDisp1^{NNN}-Shh complex (yellow) and hDisp1^{NNN}-3C-cleaved (blue). ECD1 and ECD2 move closer together, to grasp Shh. **c**, Steric clash between the Shh (purple) and the density corresponding to the uncleaved C-loop in hDisp1. The hDisp1^{NNN}-3C structure is shown in gray, while ECD1 and ECD2 are modeled in yellow and green, respectively. **d**, WT and mutant Shh-NL constructs were stably expressed in HEK293T cells. After blocking protein synthesis with

cycloheximide (100 μg/mL), Shh-NL release by purified Scube2 (1 μM) in serum-free media was measured. A single biological experiment was performed in which background-subtracted Shh-NL release was measured over five time points by NanoLuc luminescence. Initial rates were normalized to expression of each Shh-NL mutant construct, and are plotted as percentage of the rate for WT Shh-NL. Bars represent best-fit slope of a linear regression fit to the release timepoints, and error bars represent standard error of the regression fit. Adjusted p-values for pairwise ANCOVA comparison of wild type Shh-NL and each mutant are reported: *, $p < 0.05$; **, $p < 0.01$; *** $p < 0.001$; ns, not significant. Mutations are colored by their proximity to the Disp1-Shh interfaces, with yellow bars representing Shh residues close to ECD1 (N50A, L56A), green bars representing Shh residues close to ECD2 (E71A, and K74A), and grey bars representing Shh residues at unrelated sites (P26A, R61A, Q100A, and Q100H). Mutations close to ECD1 and ECD2 have the most significant defects in Shh release, while those located more distantly have non-significant or weakly significant defects. **e**, Scube-dependent Shh release is promoted only poorly by dDisp. Wild type hDisp1 or dDisp were stably co-expressed with Shh in Disp1-null HEK293T cells. The cells were then transfected with HA-tagged Scube2 or mock transfected (negative control), followed by incubation in serum-free media. Shh release after 24 hours was assayed by Western blotting. Activity of dDisp is much lower than that of hDisp1. Source data for **d** and **e** are provided as a Source Data file.

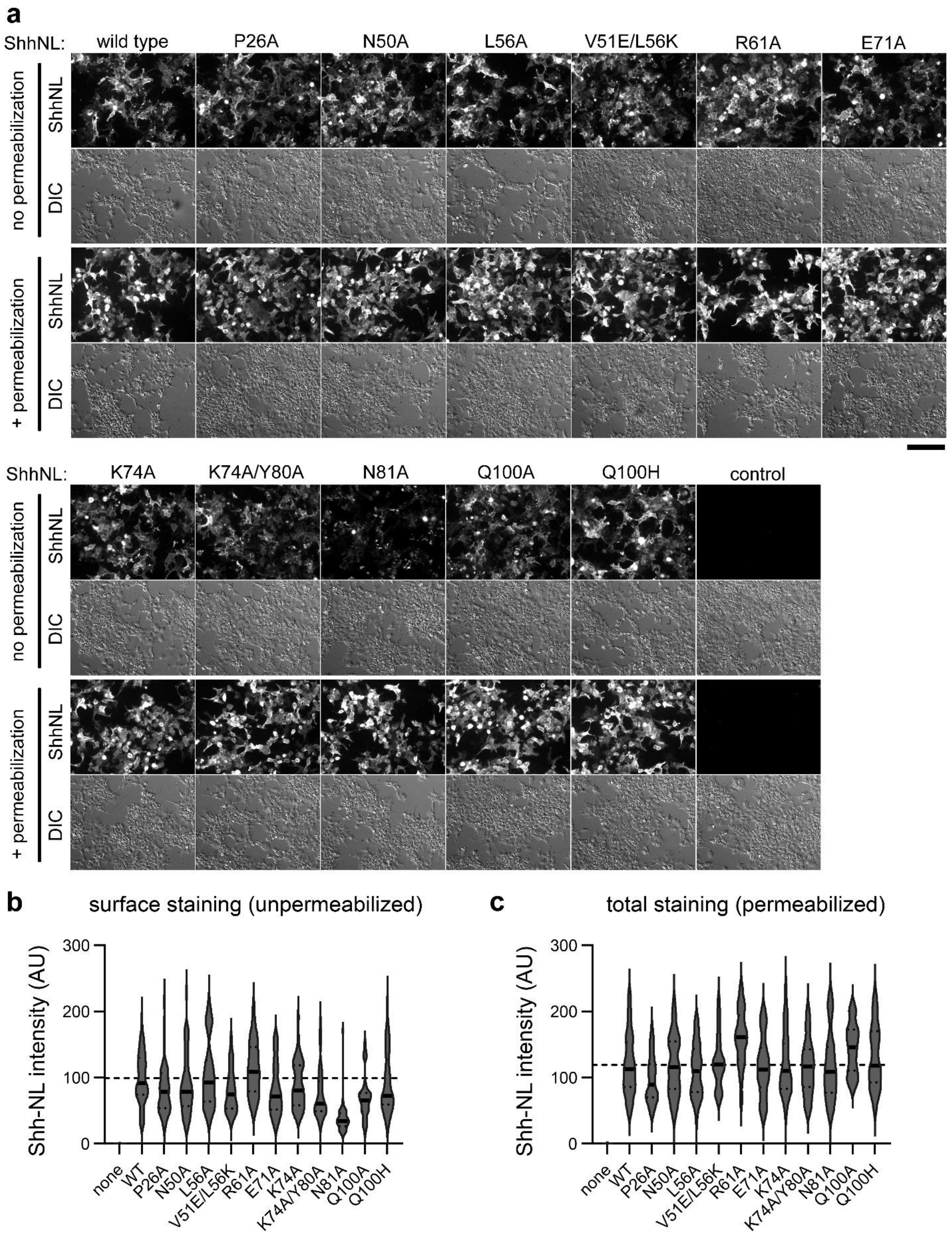

Supplementary Fig. 10 | Subcellular localization of NanoLuc-tagged Shh mutants. a,

HEK293T cells expressing wild-type or mutant Shh tagged with NanoLuc (Shh-NL) were imaged by immunofluorescence with anti-NanoLuc antibodies, with or without detergent permeabilization, to stain total Shh-NL or only Shh-NL localized to the cell surface, respectively. Cells expressing a secreted HaloTag construct served as negative control. Representative images from two independent experiments are shown (scale bar 50 microns). All constructs have similar expression levels, and all constructs except the Shh-N81A mutant have similar levels of cell surface localization. The level of Shh-N81A on the cell surface is lower, suggesting impaired trafficking to the plasma membrane, possibly due to misfolding. **b, c,** Quantification of total and cell surface staining for the experiment in (a). Staining for each Shh-NL variant is represented as a violin plot, showing the distribution of background-subtracted intensity for 40 circular regions of interest, drawn over cells. Median is represented by a thick line, and quartiles are represented by dotted lines. Source data for **b** and **c** are provided as a Source Data file.

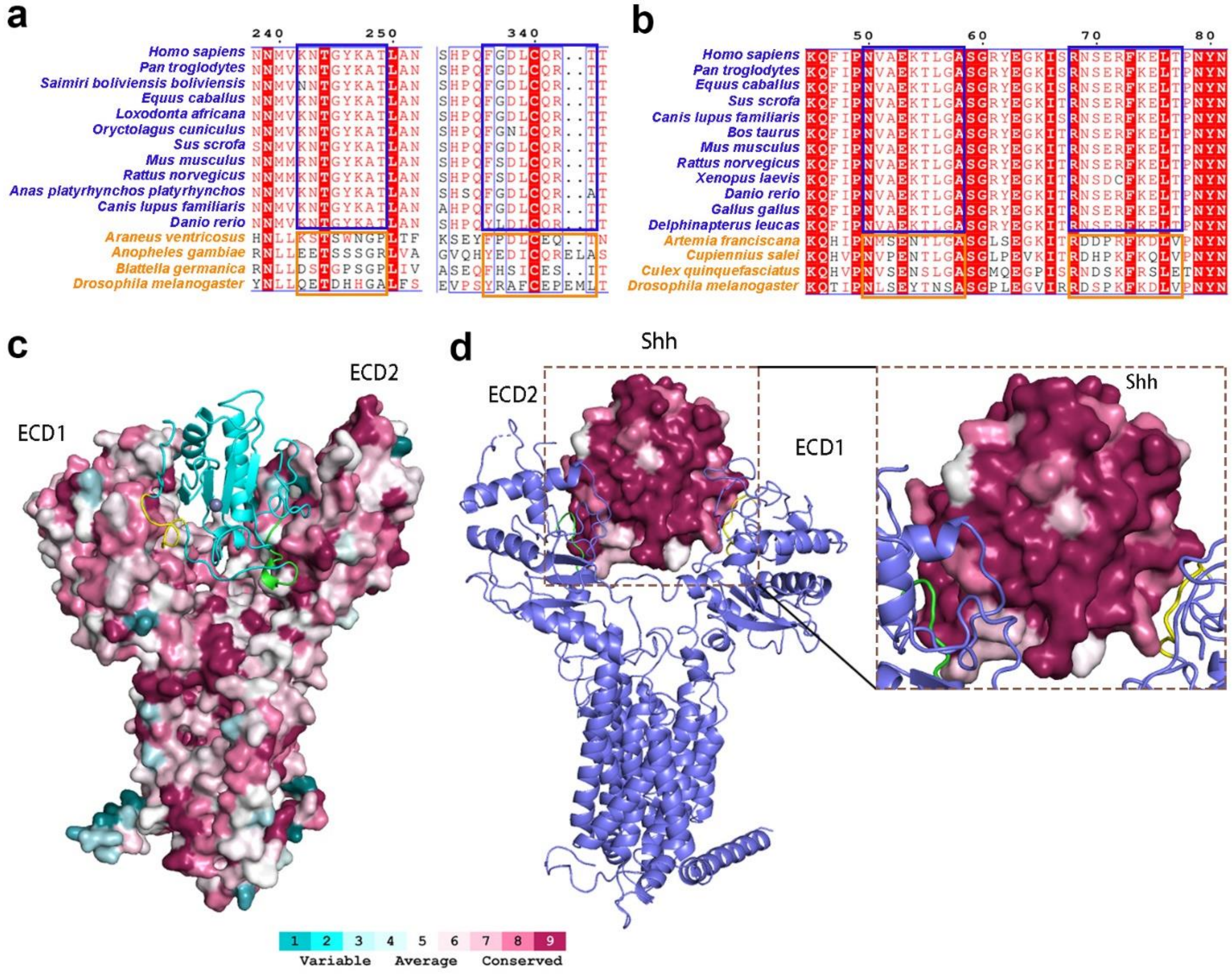

Supplementary Fig. 11 | Sequence conservation at the hDisp1-Shh interface. **a**, Sequence alignment of Disp from 12 vertebrates and 4 invertebrates around the hDisp1-Shh interface, highlighting the evolutionary divergence of the Disp at the hDisp1-Shh interface. Vertebrate Disp1 proteins (blue) have a high degree of conservation of the residues close to the interface (outlined in blue boxes), while invertebrate Disp proteins (orange) have different conserved residues (outlined in orange boxes). **b**, Sequence alignment of Shh/Hh from 12 vertebrates and 4 invertebrates around the hDisp1-Shh interface, highlighting the evolutionary divergence of the Shh/Hh at the hDisp1-Shh interface. Vertebrate Shh proteins (blue) have a high degree of conservation of the residues close to the interface (outlined in blue boxes), while invertebrate Hh

proteins (orange) have slightly different conserved residues (outlined in orange boxes). **c**, Sequence conservation of the 12 vertebrate Disp1 and 4 invertebrate Disp (sequence identity from 98% to 34%) mapped onto the hDisp1-Shh structure. The Shh residues around the hDisp1-Shh interface are highlighted in yellow (residues 50-58) and green (residues 68-77), respectively. **d**, Sequence conservation of the 12 vertebrate Shh and 4 invertebrate Hh (sequence identity from 99% to 44%) mapped onto the hDisp1-Shh structure. The hDisp1 residues around the hDisp1-Shh interface are highlighted in yellow (residues 336-343) and green (residues 242-249), respectively. The sequence conservation analysis in (**c**) and (**d**) was performed using the ConSurf Server.

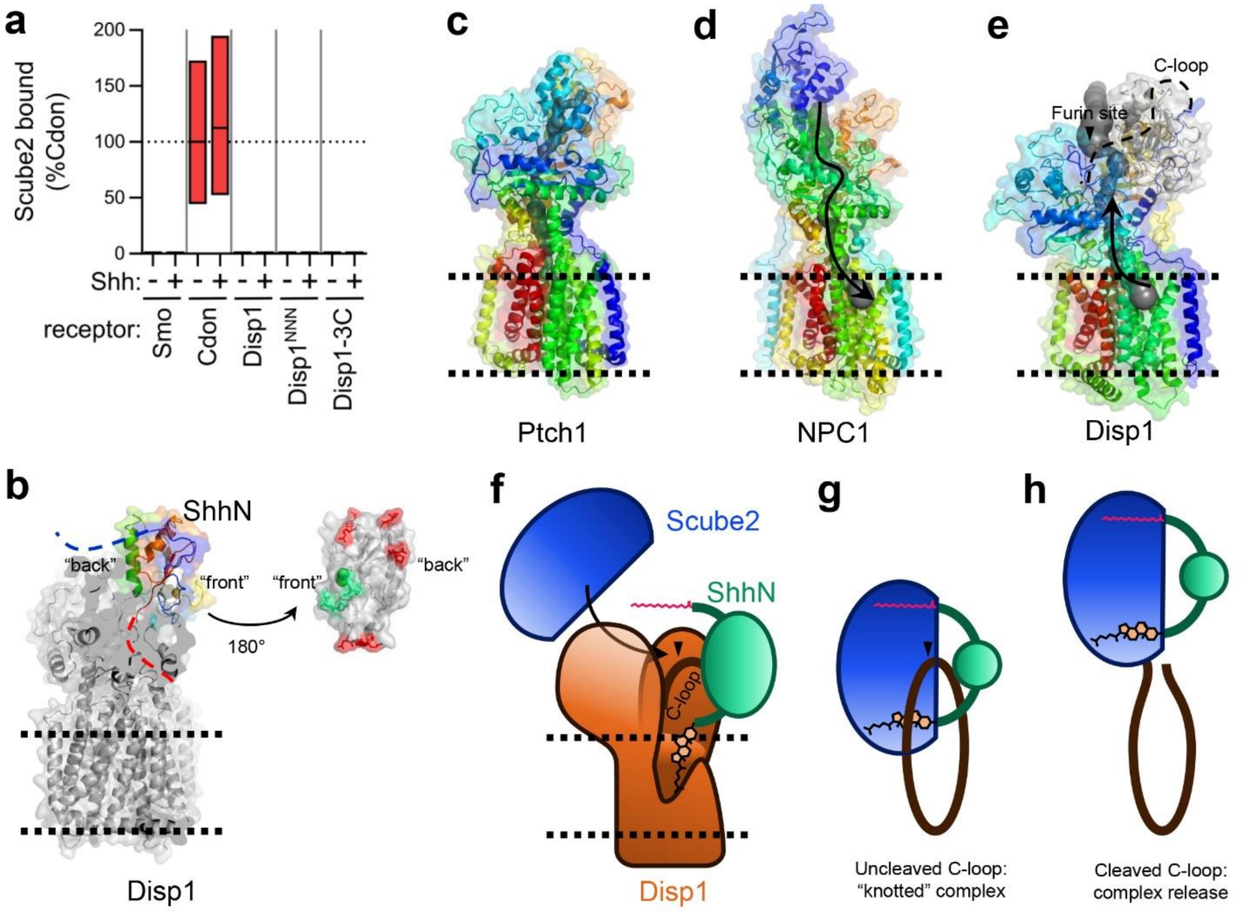

Supplementary Fig. 12 | Possible models for Shh release by Disp1. **a**, Scube2 does not stably bind Disp1, whether Shh is present or not. HEK293T cells, with or without stable Shh expression, were transiently transfected with various mCherry-tagged Disp1 constructs. Transfection with mCherry-tagged Cdon and Smo served as positive and negative control, respectively (ref 16, Wierbowski et al., 2020). The cells were incubated with purified Flag-tagged Scube2, followed by fixation and immunofluorescent staining with Alexa488-labeled anti-Flag antibody (ref 16, Wierbowski et al., 2020). Cells positive for mCherry were segmented, and bound Scube2 was measured by quantifying the Alexa488 fluorescence signal. Box plots represent median, and first and third quartiles of bound Scube2 signal. Data are normalized between background Scube2 binding to the negative control Smo, and Scube2 binding to the positive control Cdon (100%). The

number of cells that were analyzed per condition are: 355, 449, 1707, 1674, 2102, 2092, 1104, 420, 2022, and 1931. **b**, Schematic of the current hDisp1-Shh complex viewed from the side. Note the "back" and "front" surfaces of Shh, which are both solvent-exposed. Scube might interface with the "back" surface of Shh, where the N-terminal lipidated peptide appears points in the current structure. Alternatively, Scube might interface with the "front" surface of Shh, where the C-terminal lipidated peptide points in the current structure. Insertion of NanoLuc luciferase (NL) into the "front" surface of Shh does not block Scube-dependent Shh release, in contrast to insertion into the "back" surface. Results of NL insertions into Shh from (ref 15, Petrov et al., 2020) were mapped onto Shh, as shown in the inset at right. **c**, Cholesterol path in Ptch1 (PDB 6MG8). **d**, In NPC1 (PDB 6W5S), cholesterol moves from the luminal N-terminal domain (NTD, blue) to the membrane. The cholesterol transport path is shown in grey, for illustrative purpose. **e**, Speculative path for Shh cholesteryl moiety transport in Disp1. **f**, Unlike cholesterol in Ptch1 and NPC1, the sterol substrate of Disp1 is attached to Shh, which is too large to move under the C-loop. **g**, If Disp1 transported the Shh cholesteryl moiety under the C-loop and then passed it to Scube, Scube-Shh would end up entangled with Disp1. **h**, Furin cleavage of the C-loop provides a topological break, allowing free passage of the Shh C-terminal cholesteryl moiety and thus dissociation of the Scube-Shh complex from Disp1. Source data for **a** are provided as a Source Data file.

Supplementary Table 1 | Cryo-EM data collection, 3D reconstruction and model statistics.

	hDisp1^{NNN}-3C<br>EMDB-30956<br>PDB 7E2G	hDisp1^{NNN}-3C-cleaved<br>EMDB-30957<br>PDB 7E2H	hDisp1^{NNN}-ShhN<br>EMDB-30958<br>PDB 7E2I
**Data collection and processing**			
Magnification	130,000	130,000	105,000
Voltage (kV)	300	300	300
Electron exposure (e^-/Å^2)	50	50	50
Defocus range (μm)	-1.0 to -2.0	-1.0 to -2.0	-1.2 to -2.0
Pixel size (Å)	1.08	1.08	1.114
Symmetry imposed	C1	C1	C1
Initial particle image (No.)	3,788,418	1,023,748	3,773,427
Final particle image (No.)	159,333	63,043	173,169
Map resolution (Å)	3.61	3.68	4.07
FSC threshold	0.143	0.143	0.143
Map resolution range (Å)	3.5-6.0	3.5-6.0	3.6-6.0
**Refinement**			
Initial model used (PDB code)	6DMB		
Model resolution (Å)	3.61	3.68	4.07
FSC threshold	0.143	0.143	0.143
Map sharpening *B* factor (Å^2)	-140	-160	-160
Model composition			
Non-hydrogen atoms	7381	7297	8574
Protein residues	901	907	1058
Ligands	11 CHS<br>4 NAG	8 CHS<br>4 NAG	7 CHS<br>4 NAG<br>1 Zn^{2+}
B factors (Å^2)			
Protein	88.59	91.46	138.19
Ligand	91.57	97.98	164.61
R.m.s. deviations			
Bond lengths (Å^2)	0.005	0.004	0.006
Bond angles (°)	0.703	0.692	0.805
**Validation**			
MolProbity score	2.11	1.98	2.41
Clashscore	11.22	8.74	20.74
Poor rotamers (%)	0.14	0	0
Ramachandran plot			
Favored (%)	90.24	91.42	88.24
Allowed (%)	9.76	8.36	11.66
Disallowed (%)	0	0.22	0.1

Supplementary Table 2 | Primers used in the study

Primer	Sequence (5'-3')
Disp1-pCAG-1-NotI-F	ATAAGAATGCGGCCGCATGGCCATGTCCAACGGTAACAAC
Disp1-pCAG-1524-XhoI-R	CCGCTCGAGCAGAGTCTTGATCAGCAGGGAC
Disp1-D572N/D573N-F	GTGGGTATCGGTGCTAACAACGCTTTTGTGCTGTGC
Disp1-D572N/D573N-R	GCACAGCACAAAAGCGTTGTTAGCACCGATACCCAC
Disp1-D1051N-F	GTGGGTCTGTCCGTGAATTTCGCTGTGCACT
Disp1-D1051N-R	AGTGCACAGCGAAATTCACGGACAGACCCAC
Disp1-3C-F	TTCTGTTCCAGGGTCCGGGAAGCGAAGTGGACTGGAAC
Disp1-3C-R	GGACCCTGGAACAGAACTTCCAGGGATGAAGCCTGCTCGTCAGC
Shh-pCAG-1-NotI-F	ATAAGAATGCGGCCGCATGTTGCTGCTGGCTCGTTGTTTGC
Shh-pCAG-462-XhoI-R	CCGCTCGAGTCAGCTGCTTTTCACGGCCATAC
