# Supplementary material for: Structural insights into proteolytic activation of the human Dispatched1 transporter for Hedgehog morphogen release
Source: Nat Commun. 2021 Nov 29;12:6966. doi: 10.1038/s41467-021-27257-w (PMC8630017; doi:10.1038/s41467-021-27257-w)
Supplement: Supplementary file 2 — Reporting Summary [file 41467_2021_27257_MOESM2_ESM.pdf]

nature research

Corresponding author(s): Adrian Salic and Xin Gong

Last updated by author(s): Oct 24, 2021

Reporting Summary

Nature Research wishes to improve the reproducibility of the work that we publish. This form provides structure for consistency and transparency in reporting. For further information on Nature Research policies, see our Editorial Policies and the Editorial Policy Checklist.

Statistics

For all statistical analyses, confirm that the following items are present in the figure legend, table legend, main text, or Methods section.

n/a	Confirmed	
☐	☒	The exact sample size (*n*) for each experimental group/condition, given as a discrete number and unit of measurement
☐	☒	A statement on whether measurements were taken from distinct samples or whether the same sample was measured repeatedly
☐	☒	The statistical test(s) used AND whether they are one- or two-sided<br>*Only common tests should be described solely by name; describe more complex techniques in the Methods section.*
☒	☐	A description of all covariates tested
☐	☒	A description of any assumptions or corrections, such as tests of normality and adjustment for multiple comparisons
☐	☒	A full description of the statistical parameters including central tendency (e.g. means) or other basic estimates (e.g. regression coefficient) AND variation (e.g. standard deviation) or associated estimates of uncertainty (e.g. confidence intervals)
☐	☒	For null hypothesis testing, the test statistic (e.g. *F*, *t*, *r*) with confidence intervals, effect sizes, degrees of freedom and *P* value noted<br>*Give P values as exact values whenever suitable.*
☒	☐	For Bayesian analysis, information on the choice of priors and Markov chain Monte Carlo settings
☒	☐	For hierarchical and complex designs, identification of the appropriate level for tests and full reporting of outcomes
☒	☐	Estimates of effect sizes (e.g. Cohen's *d*, Pearson's *r*), indicating how they were calculated

Our web collection on statistics for biologists contains articles on many of the points above.

Software and code

Policy information about availability of computer code

Data collection	Cryo-EM data were collected with SerialEM 3.7.0; luciferase measurements were made with a Wallac VICTOR3 microplate reader and Wallac 1420 software (version 3.00 revision 2); epifluorescence microscopy images were acquired with MetaMorph software (7.10.3)
Data analysis	MotionCor2-1.1.0; Gctf_V1.18; RELION 3.0; Coot-0.8.9; Phenix-1.13; Pymol-2.2; Chimera-1.13; Clustal Omega; ESPript; ConSurf; cell-based binding data were analyzed in Matlab 2021a; Shh immunofluorescence images were analyzed in Fiji (v1.53c); release assays and microscopy images were analyzed in Microsoft Excel 2016 and GraphPad Prism 9

For manuscripts utilizing custom algorithms or software that are central to the research but not yet described in published literature, software must be made available to editors and reviewers. We strongly encourage code deposition in a community repository (e.g. GitHub). See the Nature Research guidelines for submitting code & software for further information.

Data

Policy information about availability of data

All manuscripts must include a data availability statement. This statement should provide the following information, where applicable:

- Accession codes, unique identifiers, or web links for publicly available datasets
- A list of figures that have associated raw data
- A description of any restrictions on data availability

The cryo-EM maps of hDisp1NNN-3C, hDisp1NNN-3C-cleaved and hDisp1NNN-ShhN have been deposited in the Electron Microscopy Data Bank (EMDB) with accession codes EMD-30956 [https://www.ebi.ac.uk/pdbe/entry/emdb/EMD-30956], EMD-30957 [https://www.ebi.ac.uk/pdbe/entry/emdb/EMD-30957] and EMD-30958 [https://www.ebi.ac.uk/pdbe/entry/emdb/EMD-30958], respectively. The corresponding atomic coordinates have been deposited in the Protein Data Bank (PDB) with accession codes 7E2G [http://doi.org/10.2210/pdb7E2G/pdb], 7E2H [http://doi.org/10.2210/pdb7E2H/pdb] and 7E2I [http://doi.org/10.2210/pdb7E2I/pdb], respectively. Source data are provided with this paper.

Field-specific reporting

Please select the one below that is the best fit for your research. If you are not sure, read the appropriate sections before making your selection.

☒ Life sciences ☐ Behavioural & social sciences ☐ Ecological, evolutionary & environmental sciences

For a reference copy of the document with all sections, see nature.com/documents/nr-reporting-summary-flat.pdf

Life sciences study design

All studies must disclose on these points even when the disclosure is negative.

Sample size	No statistical method was used to determine sample size; sample sizes were chosen based on the standard in the field, with three independent biological replicates being chosen for experiments requiring ANOVA analysis.
Data exclusions	No data was excluded.
Replication	Each experiment was reproduced at least twice on separate occasions. Experimental findings were reliably reproduced.
Randomization	Samples were not randomized because these studies involved defined molecular reagents used in specific mechanistic assays. Randomization is not standard in this field. This study did not involve animals or human research participants.
Blinding	Blinding was not necessary because experimental conditions were well-controlled and experimental results were quantitative and did not require subjective interpretation or analysis. Blinding is not standard in this field. This study did not involve animals or human research participants.

Reporting for specific materials, systems and methods

We require information from authors about some types of materials, experimental systems and methods used in many studies. Here, indicate whether each material, system or method listed is relevant to your study. If you are not sure if a list item applies to your research, read the appropriate section before selecting a response.

Materials & experimental systems

n/a	Involved in the study	
☐	☒	Antibodies
☐	☒	Eukaryotic cell lines
☒	☐	Palaeontology and archaeology
☒	☐	Animals and other organisms
☒	☐	Human research participants
☒	☐	Clinical data
☒	☐	Dual use research of concern

Methods

n/a	Involved in the study	
☒	☐	ChIP-seq
☒	☐	Flow cytometry
☒	☐	MRI-based neuroimaging

Antibodies

Antibodies used	rabbit anti-mCherry polyclonal antibody (Nedelcu et al., 2013)<br>mouse anti-Flag monoclonal antibody (Sangon Biotech Cat# D191041)<br>mouse anti-His monoclonal antibody (Sangon Biotech Cat# D191001)<br>goat anti-mouse HRP-conjugated secondary antibody (Sangon Biotech Cat# D110087)<br>mouse anti-Strep monoclonal antibody (IBA Lifesciences Cat# 2-1507-001)<br>mouse monoclonal anti-FLAG Ml (from hybridoma ATCC Cat# HB-9259; RRID CVCL_J730)<br>mouse monoclonal anti-HPC (A.C. Kruse, Roche Cat# 11814516001; RRID:AB_390920)<br>mouse monoclonal anti-tubulin, clone DMlA (Sigma Cat# T6199; RRID: AB_ 477583)<br>mouse monoclonal anti-NanoLuc (Promega Cat# N7000)<br>rabbit monoclonal anti-SHH, clone C9C5 (Cell Signaling Technology Cat# 2207S; RRID: AB_2188191)<br>rat anti-HA, clone 3F10–HRP conjugate (Roche Cat# 12013819001; RRID: AB_390917)<br>sheep anti-mouse lgG–HRP conjugate (Jackson ImmunoResearch Cat# 515-005-003; RRID: AB_2340287)<br>donkey anti-rabbit lgG–HRP conjugate (GE Healthcare Cat# NA934; RRID: AB_772206)<br>goat anti-mouse lgG–Alexa Fluor 594 conjugate (Thermo Cat# A-11032)
Validation	anti-mCherry: Nedelcu et al., 2013<br>anti-Flag: Anti-Flag tag mouse monoclonal antibody recognizes the DYKDDDDK peptide fused to either the amino- or carboxy-terminus of targeted proteins, validated for Western blotting.<br>anti-His: Anti-6×His Tag mouse monoclonal antibody recognizes the His-tag fused to targeted proteins in transfected or transformed cells, validated for Western blotting.

anti-Strep: murine monoclonal antibody to detect Strep-tag®II and Twin-Strep-tag® fusion proteins, validated for Western blotting.
anti-FLAG MI: US Patent Number 4,851,341
anti-HPC: US Patent Number 7,247,453B1
anti-tubulin: Blose et al., 1984
anti-NanoLuc: Anti-NanoLuc® Monoclonal Antibody (Cat.# N7000) is a protein A/G affinity-purified mouse monoclonal antibody that is used to detect NanoLuc® luciferase or NanoLuc® fusion proteins via Western blotting. When Anti-NanoLuc® Monoclonal Antibody was used in Western blots, no cross-reactivity with the LgBiT subunit of NanoBiT® technology was observed. Validation for immunofluorescence detection can be found in Extended Data Figure 10.
anti-SHH: monoclonal antibody raised against synthetic peptide from human SHH; predicted to detect all vertebrate SHH orthologs; does not detect SHH paralogs (IHH and DHH); validated for Western blotting
anti-HA HRP conjugates: recognizes 9-aa sequence YPYDVPDYA, derived from the human influenza hemagglutinin (HA) protein anti-mouse IgG HRP conjugate: Based on immunoelectrophoresis and/or ELISA, the antibody reacts with whole molecule mouse IgG. It also reacts with the light chains of other mouse immunoglobulins. No antibody was detected against non-immunoglobulin serum proteins. The antibody may cross-react with immunoglobulins from other species.
anti-rabbit IgG HRP conjugate: Highly species-specific.
anti-mouse IgG Alexa Fluor 594 conjugate: To minimize cross-reactivity, these goat anti-mouse IgG (H+L) whole secondary antibodies have been affinity purified and cross-adsorbed against bovine IgG, goat IgG, rabbit IgG, rat IgG, human IgG, and human serum. Validated for immunofluorescence microscopy.

Eukaryotic cell lines

Policy information about cell lines

Cell line source(s)	HEK293F (Thermo Fisher Scientific); HEK293T (ATCC Cat# CRL-3216; RRID: CVCL_0063); MGATI -/- HEK293S (ATCC Cat# CRL-3022; RRID: CVCL_A785)
Authentication	No
Mycoplasma contamination	No
Commonly misidentified lines (See ICLAC register)	No
