# Supplementary material for: Structural insights into proteolytic activation of the human Dispatched1 transporter for Hedgehog morphogen release
Source: Nat Commun. 2021 Nov 29;12:6966. doi: 10.1038/s41467-021-27257-w (PMC8630017; doi:10.1038/s41467-021-27257-w)

Fig. 1

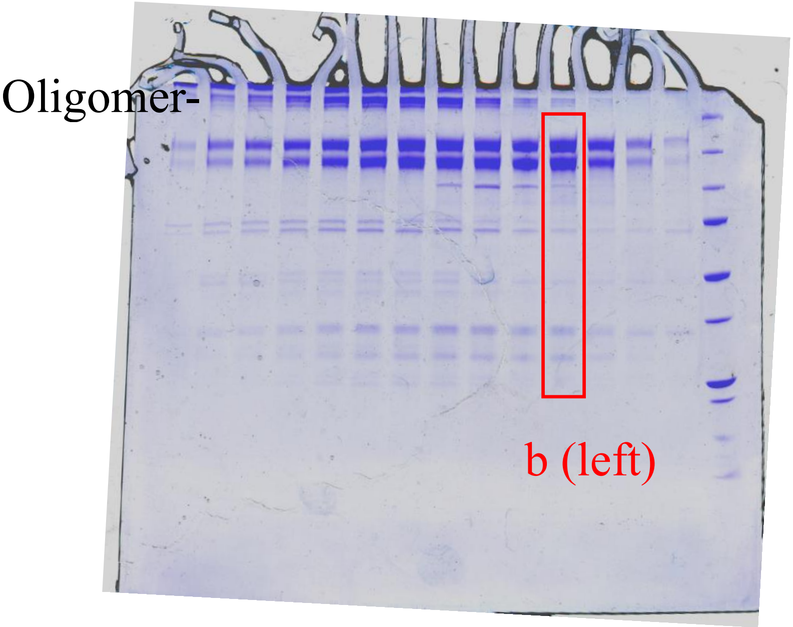

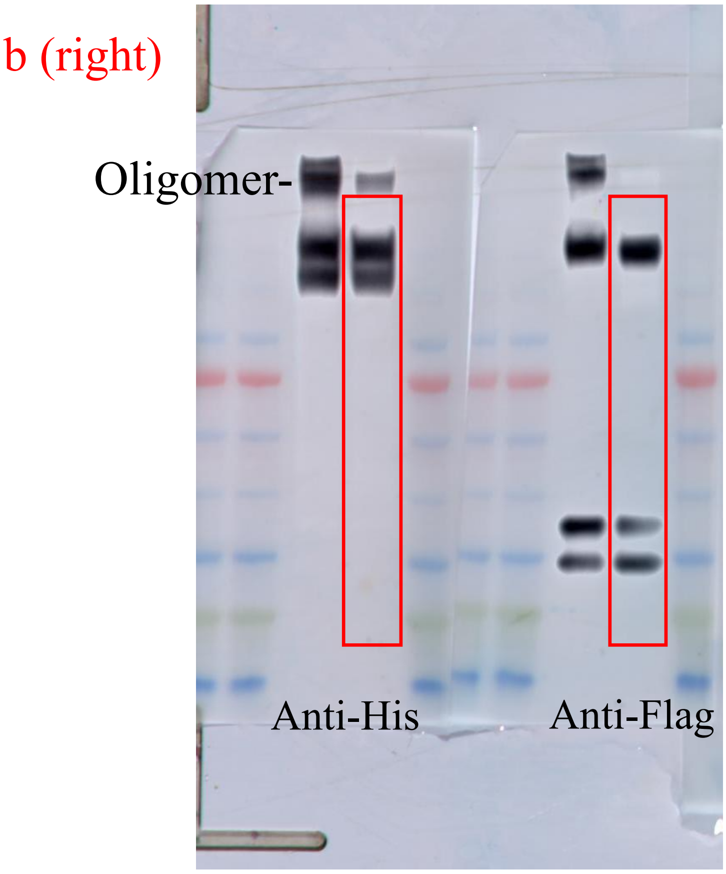

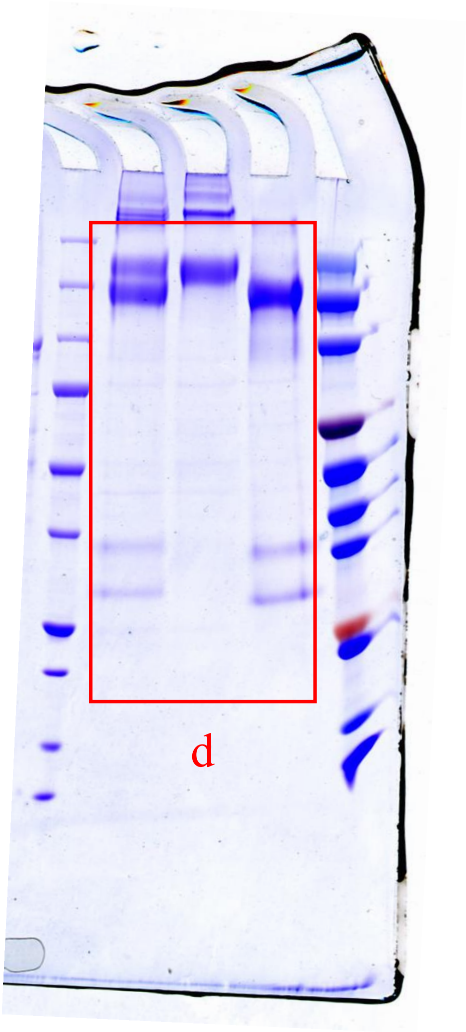

g

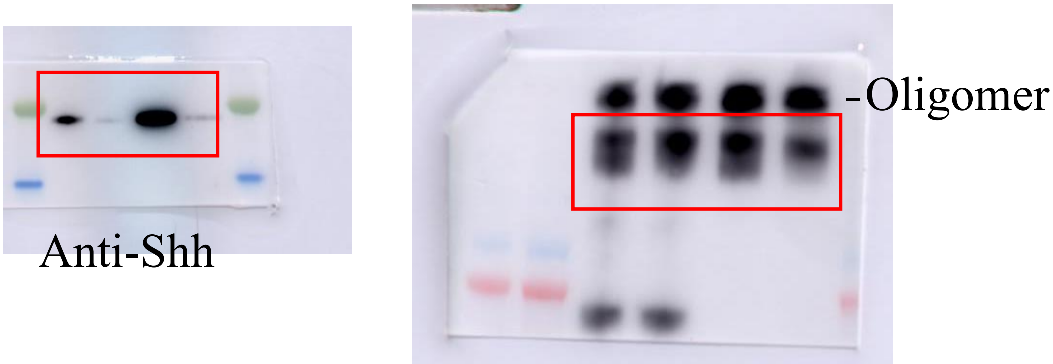

Anti-strep

Fig. 1f

anti-mCherry

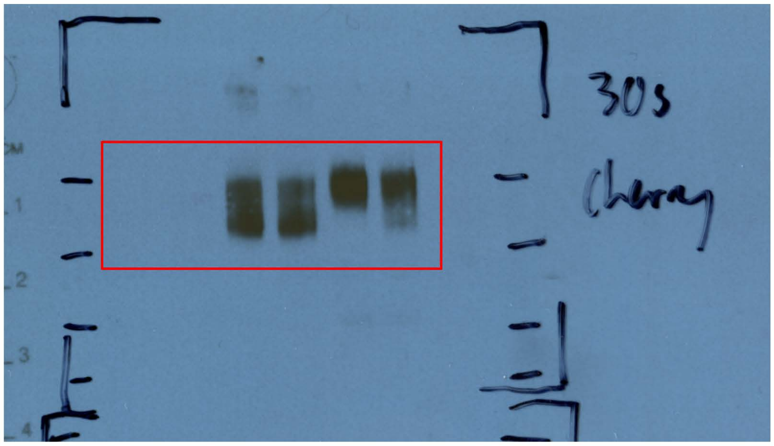

anti-HPC
(top band)

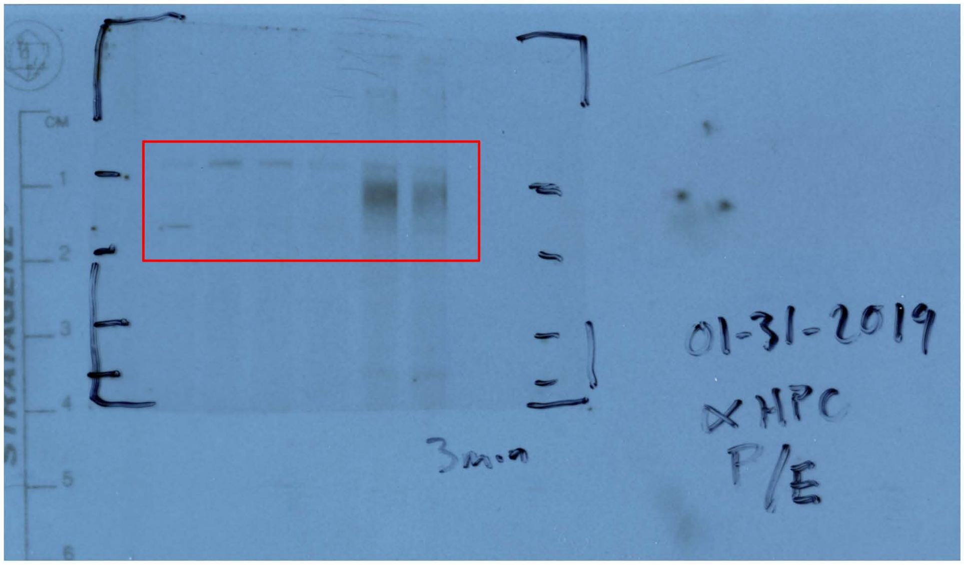

anti-HPC
(bottom band)

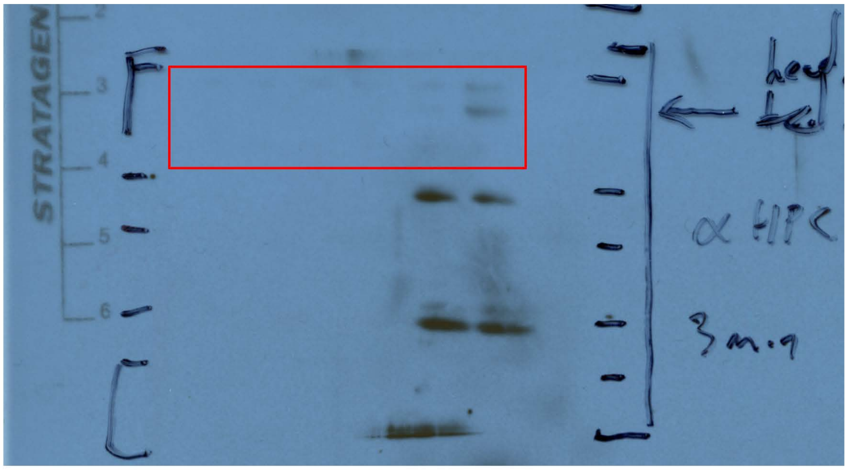

anti-tubulin

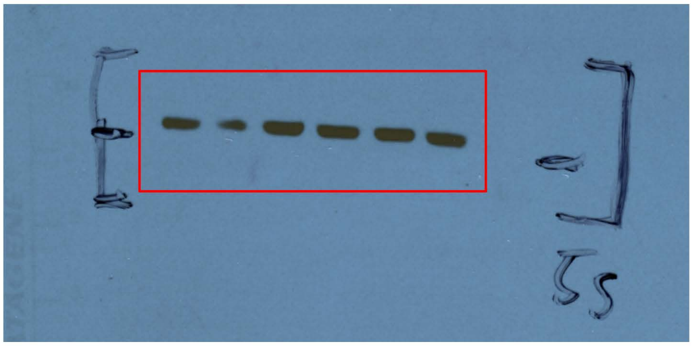

Fig. 2d

anti-mCherry (lysate)

anti-tubulin (lysate)

anti-Shh (lysate)

anti-HA (media)

anti-Shh (media)

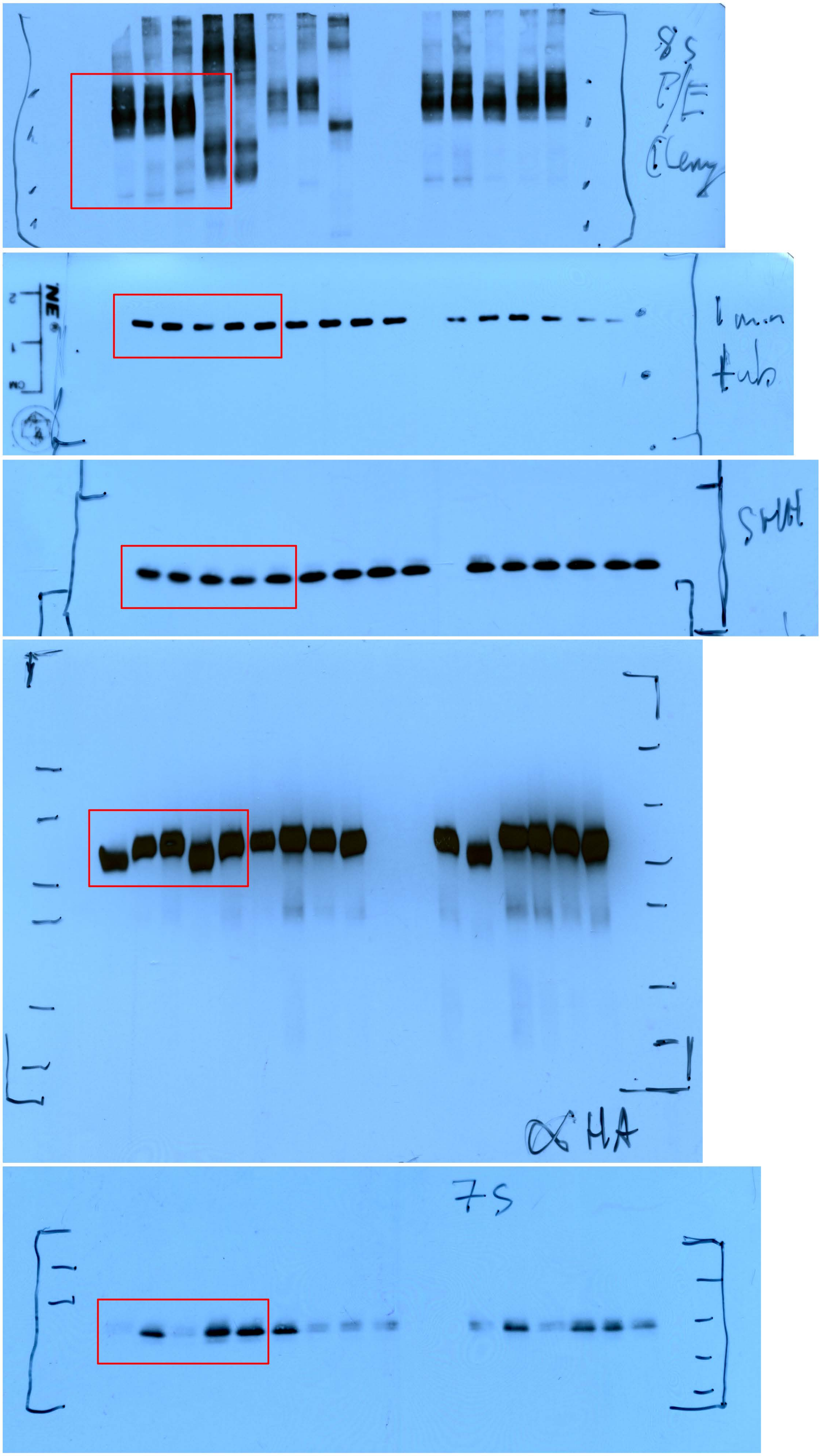

Supplementary Fig. 1

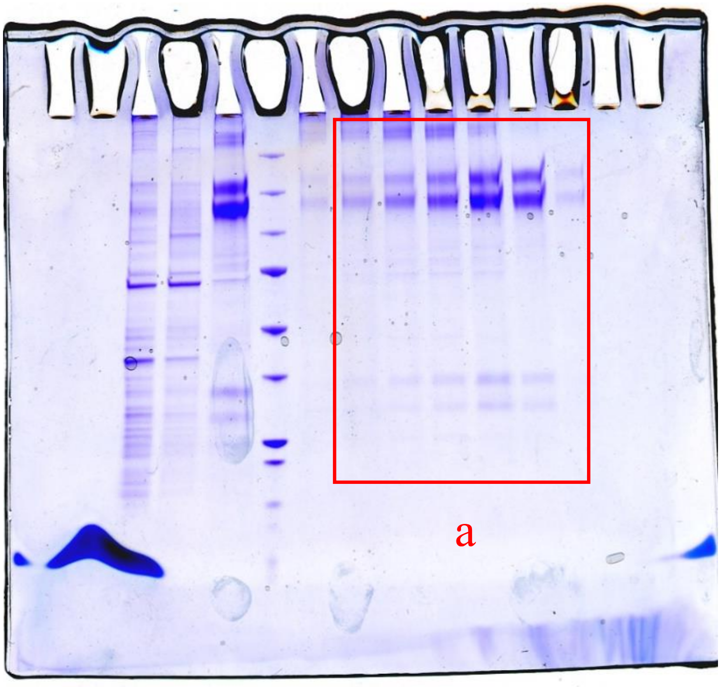

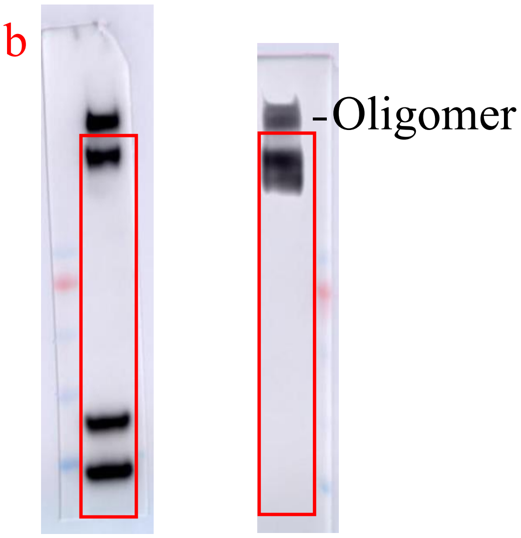

Anti-flag Anti-His

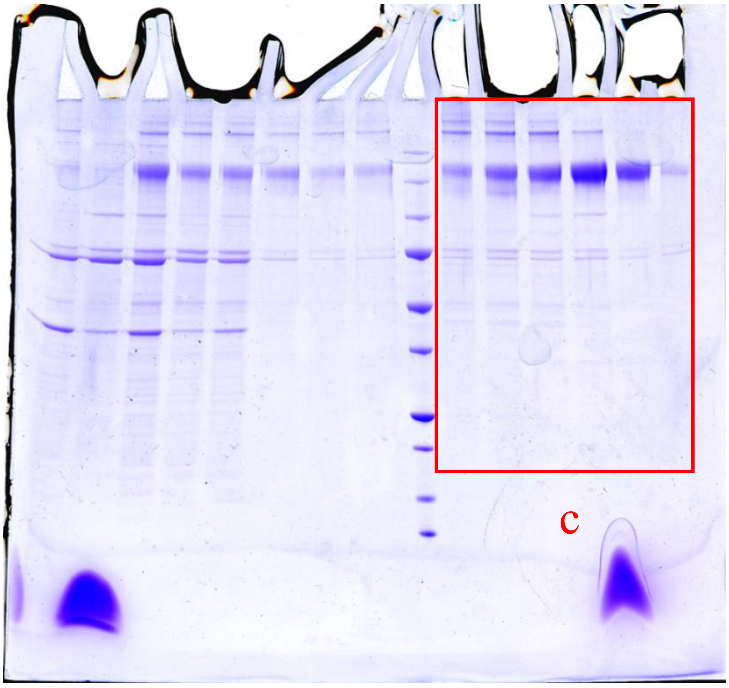
c

Supplementary Fig. 4d

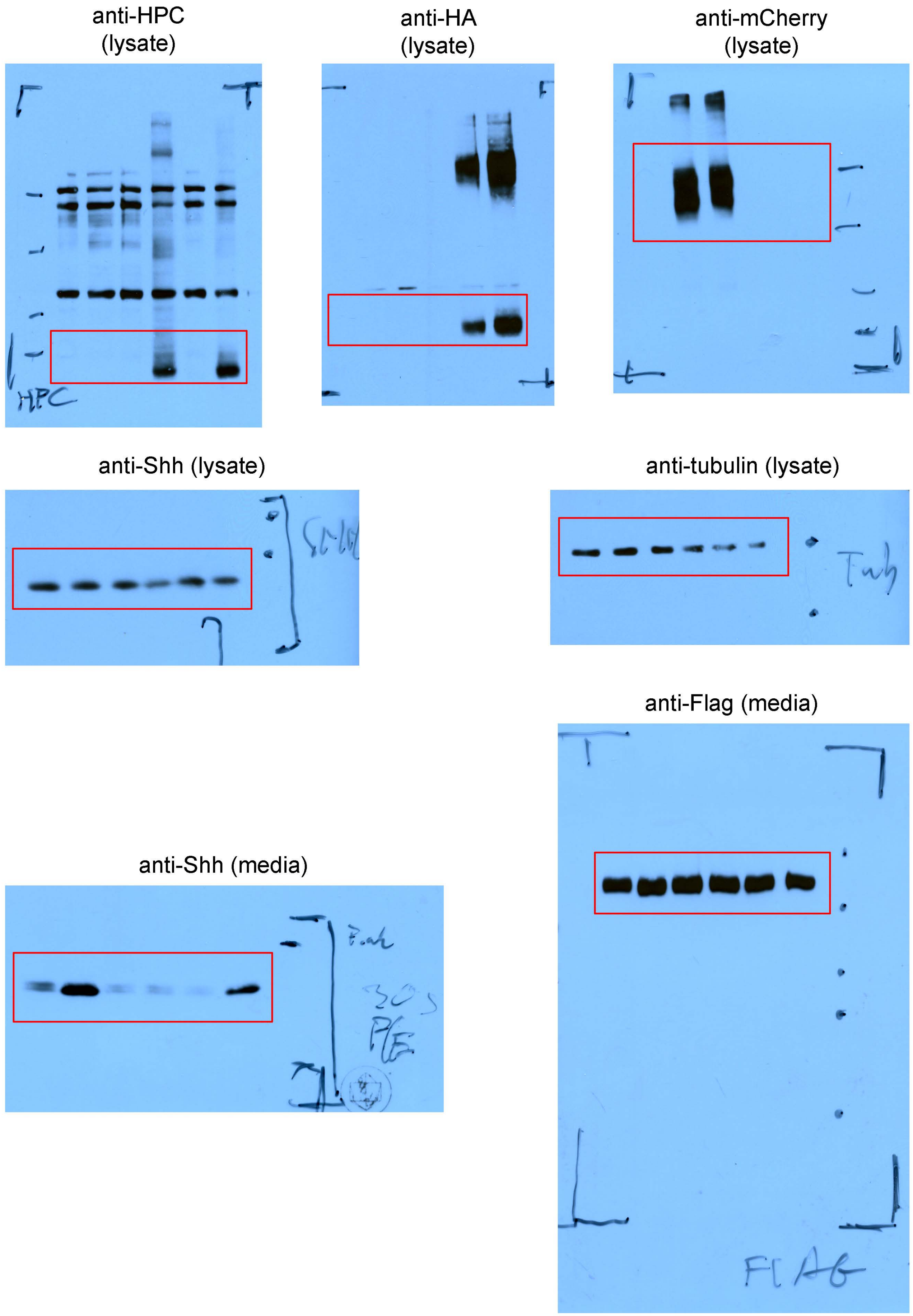

Supplementary Fig. 9e

anti-HA (media)

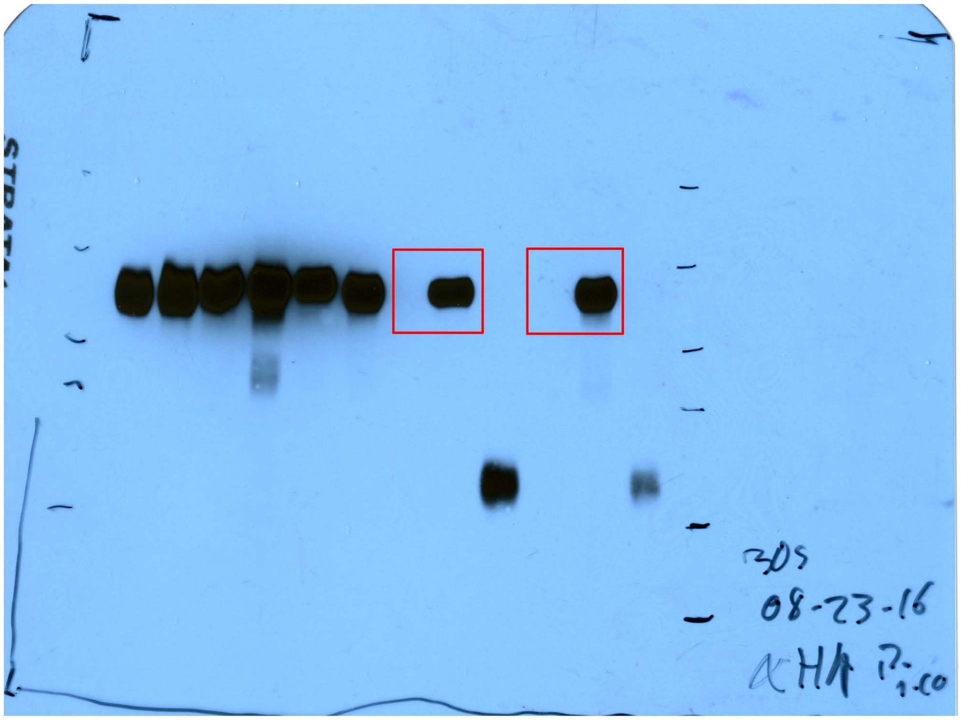

anti-Shh (media)

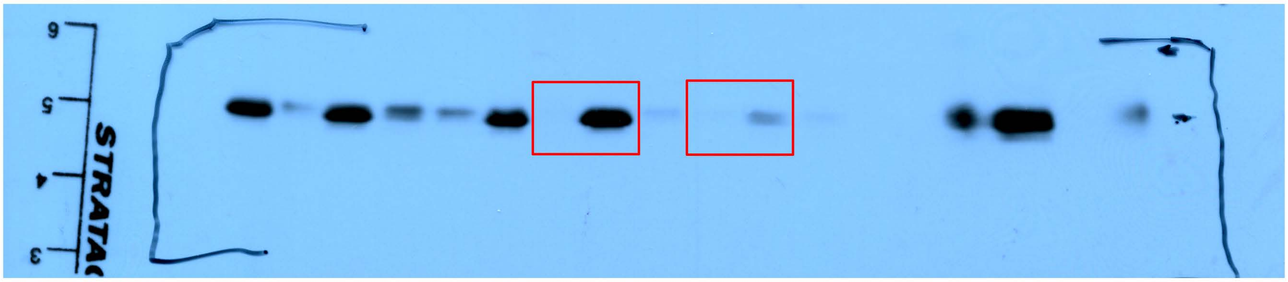

Supplement: Supplementary file 3 — Source Data [file 41467_2021_27257_MOESM3_ESM.zip › Source Data/Graphic Source Data.pdf]
